# Biochemical and structural characterization of an aromatic ring–hydroxylating dioxygenase for terephthalic acid catabolism

**DOI:** 10.1073/pnas.2121426119

**Published:** 2022-03-21

**Authors:** William M. Kincannon, Michael Zahn, Rita Clare, Jessica Lusty Beech, Ari Romberg, James Larson, Brian Bothner, Gregg T. Beckham, John E. McGeehan, Jennifer L. DuBois

**Affiliations:** ^a^Department of Biochemistry, Montana State University, Bozeman, MT 59717;; ^b^Centre for Enzyme Innovation, University of Portsmouth, Portsmouth, PO1 2PY, United Kingdom;; ^c^Bio-Optimized Technologies to Keep Thermoplastics out of Landfills and the Environment Consortium, Golden, CO 80401;; ^d^National Renewable Energy Laboratory, Renewable Resources and Enabling Sciences Center, Golden, CO 80401

**Keywords:** Poly(ethylene terephthalate), plastic, Rieske dioxygenase, terephthalate, oxygenase

## Abstract

Several bacteria possess components of catabolic pathways for the synthetic polyester poly(ethylene terephthalate) (PET). These proceed by hydrolyzing the ester linkages of the polymer to its monomers, ethylene glycol and terephthalate (TPA), which are further converted into common metabolites. These pathways are crucial for genetically engineering microbes for PET upcycling, prompting interest in their fundamental biochemical and structural elucidation. Terephthalate dioxygenase (TPADO) and its cognate reductase make up a complex multimetalloenzyme system that dihydroxylates TPA, activating it for enzymatic decarboxylation to yield protocatechuic acid (PCA). Here, we report structural, biochemical, and bioinformatic analyses of TPADO. Together, these data illustrate the remarkable adaptation of TPADO to the TPA dianion as its preferred substrate, with small, protonatable ring 2-carbon substituents being among the few permitted substrate modifications. TPADO is a Rieske [2Fe2S] and mononuclear nonheme iron-dependent oxygenase (Rieske oxygenase) that shares low sequence similarity with most structurally characterized members of its family. Structural data show an α-helix–associated histidine side chain that rotates into an Fe (II)–coordinating position following binding of the substrate into an adjacent pocket. TPA interactions with side chains in this pocket were not conserved in homologs with different substrate preferences. The binding mode of the less symmetric 2-hydroxy-TPA substrate, the observation that PCA is its oxygenation product, and the close relationship of the TPADO α-subunit to that of anthranilate dioxygenase allowed us to propose a structure-based model for product formation. Future efforts to identify, evolve, or engineer TPADO variants with desirable properties will be enabled by the results described here.

## Significance

More than 400 million tons of plastic waste is produced each year, the overwhelming majority of which ends up in landfills. Bioconversion strategies aimed at plastics have emerged as important components of enabling a circular economy for synthetic plastics, especially those that exhibit chemically similar linkages to those found in nature, such as polyesters. The enzyme system described in this work is essential for mineralization of the xenobiotic components of poly(ethylene terephthalate) (PET) in the biosphere. Our description of its structure and substrate preferences lays the groundwork for in vivo or ex vivo engineering of this system for PET upcycling.

**Fig. 1.**
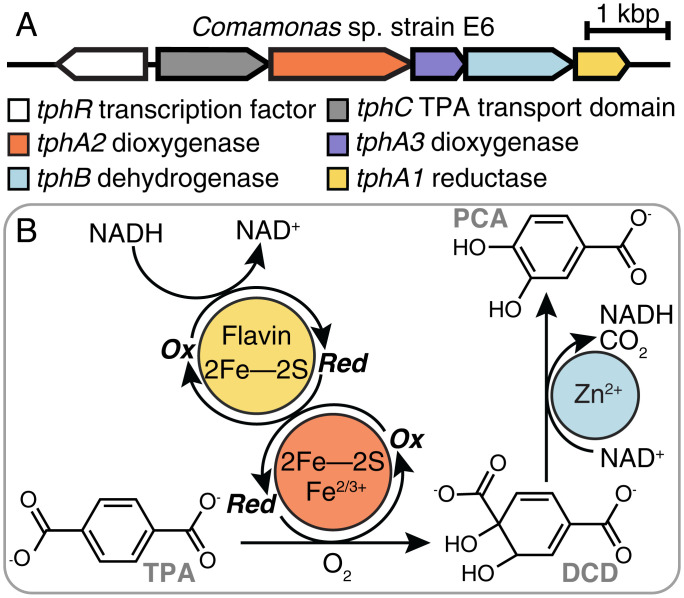
Pathway for enzymatic conversion of TPA to PCA. (*A*) Organization of the operon of genes encoding a TPA-sensing transcription factor, TPA transporter domain, dioxygenase, reductase, and dehydrogenase. (*B*) TPADO (orange) uses molecular oxygen to dihydroxylate TPA, and reducing equivalents are supplied by the reductase (yellow). The product, DCD, is converted to protocatechuate via a Zn-dependent dehydrogenase (blue).

The discovery of a bacterium that assimilates poly(ethylene terephthalate) (PET) (1, 2), the synthetic polymer used for clothing, single-use plastic bottles, and carpets, has generated great excitement over the prospect of using biological catalysis for recycling this abundant waste product (3–7). The pathway for PET assimilation by *Ideonella sakaiensis* begins with a pair of esterases that hydrolyze the polymer to its constituent monomers, ethylene glycol and terephthalate (TPA). Ethylene glycol is a natural product that is metabolized by multiple bacteria (8–10). TPA resembles plant-derived aromatic compounds, but it is not widely known as a substrate for bacterial growth. Because of its size and charge (−2 at pH 7), TPA must be actively transported into the cell, where it is *cis*-dihydroxylated and dearomatized to yield 1,2-dihydroxy-3,5-cyclohexadiene-1,4-dicarboxylate (DCD) (11–17). The initial dihydroxylation is catalyzed by an O_2_-dependent terephthalate dioxygenase (TPADO) working in conjunction with an NAD(P)H, flavin, and iron–sulfur-dependent reductase. A zinc-dependent dehydrogenase finally reductively decarboxylates DCD to produce protocatechuate (protocatechuic acid [PCA]) (Fig. 1) (1). Interest in using this pathway, either in vitro or engineered into microbes optimized for PET conversion, is motivated by the societal, ecological, and economic benefits of plastics reclamation, recycling, and upcycling (7, 18, 19). Obtaining a structural and functional understanding of each of the four enzymes is an essential step toward these future applications.

Native systems for TPA import and *cis*-dihydroxylation have thus far been identified in several bacteria, including *I. sakaiensis* and several strains that do not use PET as a carbon source. The latter includes *Comamonas* sp. strain E6 (12, 17), *Comamonas testosteroni* T-2 (15), *C. testosteroni* YZW-D (13), *Delftia tsuruhatensis* T7 (20), *Rhodococcus* sp. DK17 (11), *Rhodococcus jostii* RHA1 (14), *Pseudomonas umsongensis GO16* (8), and *Acinetobacter baylyi* ADP1 (TPA importer) (21). Several studies have introduced TPA catabolism into microbes for metabolic engineering applications as well (22–26).

The TPADO enzyme is a member of a large family of Rieske oxygenases (ROs). Several members of this family permit bacteria to aerobically assimilate and thereby, remediate a wide range of environmental contaminants (27), such as naphthalene, pyrene, toluene, and chlorobenzoate. Several of these compounds resemble natural metabolites, including benzoate, cinnamate, picolinate, and salicylate, all of which are also RO substrates (28). Naphthalene dioxygenase (NDO), a paradigmatic RO (29–31), catalyzes stereo- and regioselective dihydroxylations on a range of aromatic substrates. Other family members likewise catalyze an array of mono- and dihydroxylations, *O*- and *N*-dealkylations (28), desaturations, and sulfoxidations, where several prior studies have suggested a structural basis for reaction type or substrate preference (27, 32). ROs possess a family-defining [2Fe-2S] Rieske cluster that delivers electrons sequentially to an adjacent mononuclear nonheme iron center, where O_2_ is reductively activated. The ultimate electron source is NAD(P)H, which transfers a hydride to a flavin adenine dinucleotide cofactor in a separate reductase enzyme. One to two additional [Fe-S] clusters serve as one-electron (1e−) shuttles to the active site (*SI Appendix*, Fig. S1). These clusters are found in diverse protein domain architectures that have traditionally been used for subtyping ROs in types I through V (33). Among the type II family members, which include TPADO, sequence conservation can be surprisingly low (34). Additionally, until recently, there were no structurally characterized examples of type II enzymes. Predicting TPA-directed activity among type II ROs based solely on the primary sequence of the catalytic (α)-subunit is consequently challenging, limiting efforts to prospect for TPADO homologs in sequence databases.

In this work, we describe multiple crystal structures of the TPADO from *Comamonas* sp. strain E6 (17) in complex with both TPA and an *ortho*-substituted analog that links TPADO to salicylate 1-hydroxylase (AhdA1c) (35) and anthranilate 1,2-dioxygenase (AndAc) (36) in mechanism. Further, we define catalytic parameters and describe the specificity of TPADO for *para*-dicarboxylate anions that is well explained by the binding mode observed in the structures. Finally, we show that TPADO has functionally significant sequence relationships with aryl-carboxylate–directed Ros, which in conjunction with the structural data, can be used to propose a TPA recognition sequence motif. The results establish this TPADO and its cognate reductase as a foundation for further protein and strain engineering efforts to achieve biological upcycling of PET plastic.

## Results

### A Two-Enzyme System Dioxygenates TPA In Vitro.

TPADO, a 196-kDa complex of TphA2 and TphA3 subunits, and its cognate reductase (TphA1, 36.4 kDa) from *Comamonas* sp. strain E6 were heterologously expressed in *Escherichia coli* (*SI Appendix*, Figs. S2 and S3 and *Supplementary Methods*), with conditions then optimized to maximize both protein and cofactor yields as guided by studies from Ballou and coworkers (37). The α- and β-subunits of TPADO were coexpressed from a single isopropyl β-D-1-thiogalactopyranoside (IPTG)-inducible pET-DUET vector, where the α-subunit contained a C-terminal His_6_ tag that was used for affinity purification at yields >20 mg/L culture. Native mass spectrometry identified the α_3_β_3_-oligomer as the major component, with the individual monomers as minor constituents (*SI Appendix*, Fig. S4). The monomeric reductase was purified via a C-terminal His_6_ tag, with yields >10 mg/L culture.

Ultraviolet (UV)/visible (vis), atomic absorption, and electron paramagnetic spectroscopies confirmed the presence and type of [2Fe-2S] cluster in each protein (Rieske and plant types in TPADO and the reductase, respectively) (*SI Appendix*, Figs. S5–S7) based on their characteristic g values and absorbance maxima. Combined metal and protein analyses predicted approximately stoichiometric occupancy of the expected cofactors in TPADO; however, this assumes that all of the protein (by mass) was present as an intact α_3_β_3_-oligomer and that all iron was cofactor associated. Consequently, the concentrations of TPADO used throughout this study, reported in terms of TPADO active sites (three per α_3_β_3_-unit), are undoubtedly overestimated since at least some catalytically inactive α- and β-monomers and unpopulated cofactor binding sites were likely present.

**Fig. 2.**
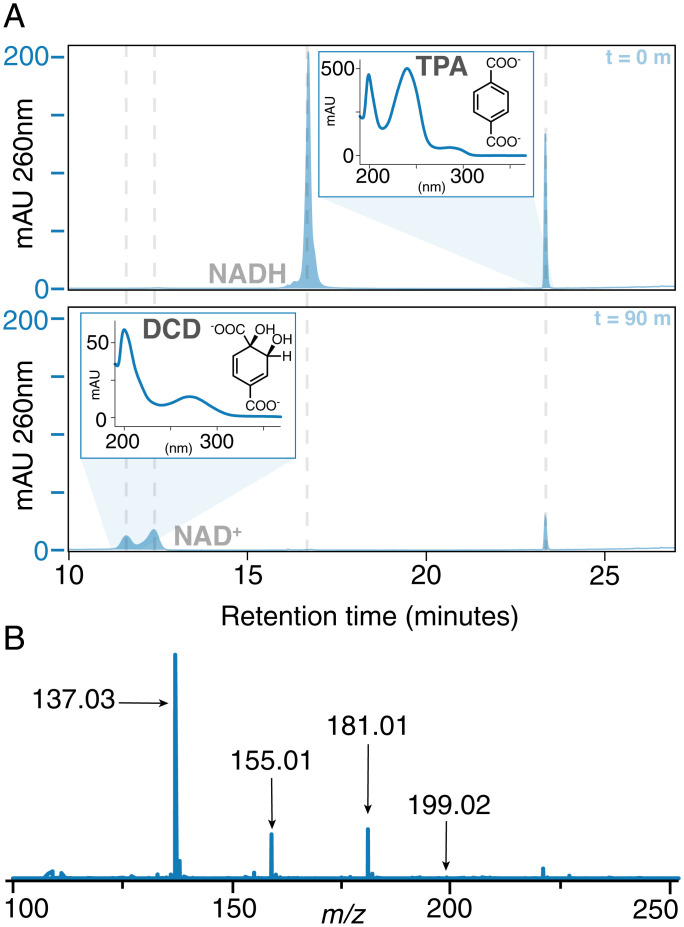
TPADO converts TPA to DCD. (*A*) HPLC chromatograms of a reaction mixture containing TPADO, its reductase, NADH, and TPA in air after 0 min (*Upper*) and 90 min (*Lower*) of reaction time. The data,measured in milliabsorbance units (mAU) versus retention time, illustrate the disappearance of NADH with the concomitant formation of new peaks at 12 min (assigned as DCD) and 13 min (NAD^+^). Peak assignments were made based on identical retention times and UV/vis spectra (*Insets*) relative to known standards (*SI Appendix*, Figs. S10 and S11), except for DCD, for which no standards were available. In that case, the product was assigned based on (*B*) its mass spectrum, where the parent ion (*m/z* = 199.02) and fragments predicted to result from loss of water (*m/z* = 181.01), CO_2_ (*m/z* = 155.01), or both (*m/z* = 137.03) were readily identified (*SI Appendix*, Fig. S8).

TPA was converted by the TPADO/reductase system to an oxidized product specifically in the presence of nicotinamide adenine dinucleotide hydride (NADH) rather than its phosphorylated analog, NADPH. The oxygenation product could be resolved from the NAD^+^ coproduct by reverse-phase high-performance liquid chromatography (HPLC) when subjected to a complex gradient (*SI Appendix*, *Supplementary Methods*). The distinctive UV/vis absorbance spectrum for the product (Fig. 2*A*) was consistent with absorbance maxima reported for DCD (15). However, this molecule is not commercially available, nor does extensive characterization exist in published literature. Here, we chromatographically resolved the product from NAD^+^ by HPLC and obtained its UV/vis absorbance spectrum and mass spectral (MS) analyses. Under the ionization conditions used, DCD exhibited fragmentation consistent with the loss of CO_2_ and/or H_2_O (Fig. 2*B*). The expected exact mass of the intact, positively ionized DCD has a molecular ion peak with mass/charge (*m/z*) = 199, while the predominant ion observed at the retention time of the product had an observed *m/z* = 137. TPA also displayed similar behavior, losing CO_2_ during source ionization (*SI Appendix*, Fig. S8). Targeted fragmentation of the weak-intensity, intact DCD and TPA peaks by liquid chromatography coupled to tandem mass spectrometry (LC-MS-MS) yielded fragmentation profiles that were consistent with the observed source ionization fragmentation, supporting the definitive identification of the latter compound.

Steady-state kinetic parameters were measured as a function of variable [TPA] in ambient air (*SI Appendix*, Fig. S9). The data readily fit the Michaelis–Menten model, yielding *k*_cat_ (apparent) = 12 ± 0.3 min^−1^ and *K*_M_[TPA] (apparent) = 9.6 ± 1 μM. The parameters are apparent as saturating concentrations for NADH and O_2_ have not been determined, and *k*_cat_ (maximal velocity × [enzyme]^−1^) is likely underestimated. The *k*_cat_ and low-micromolar *K*_M_ are nonetheless similar to values measured for the TPADO homolog salicylate 5-monooxygenase (NagGH) from *Ralstonia* sp. strain U2 (*k*_cat_ = 7.81 min^−1^, *K*_M_ = 22.4 μM) (38), suggesting that TPADO is well adapted to its substrate, although slow in absolute terms. A total turnover number (TTN) of 704 (per apparent TPADO active site) was measured for the system via determination of unreacted TPA by HPLC when all other reactants were in excess. This TTN is low compared with several enzymes used in applied work, potentially due to overcounting of intact metallo-active sites here or to intrinsic instability of the enzymes. Either explanation suggests the need for engineered improvements to enzyme stability for future applications (39).

### TPADO Exhibits Remarkable Specificity for *Para*-Dicarboxylates.

**Scheme 1.**
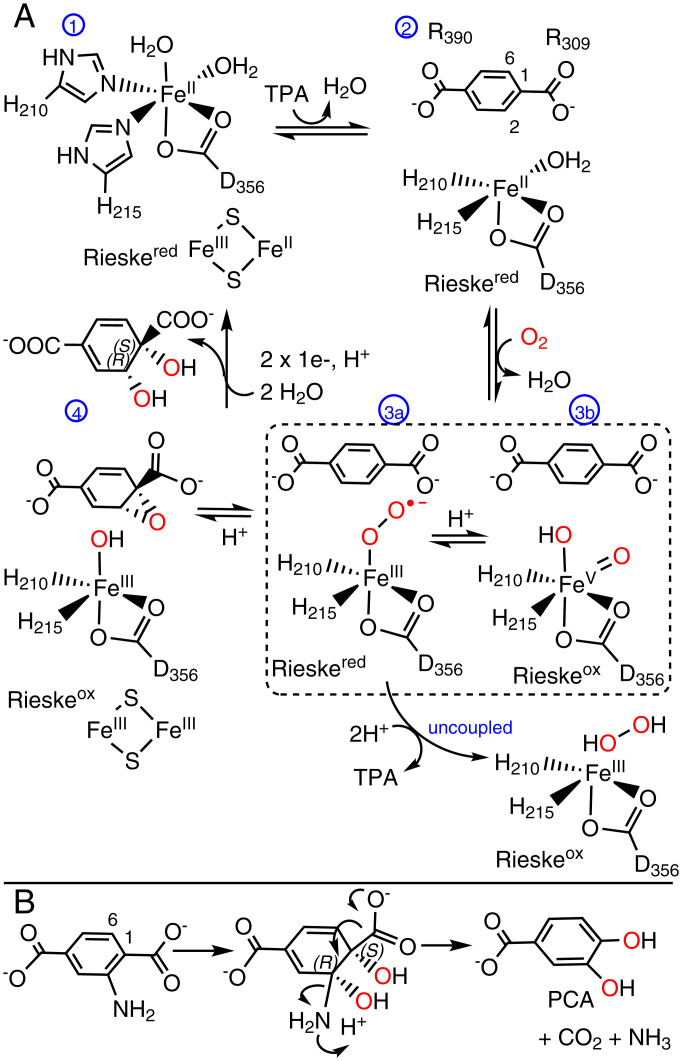
A canonical mechanism for ROs adapted for TPADO. (*A*) The fully reduced, resting active site (**1**) has a ferrous hexacoordinate mononuclear iron and reduced Rieske cluster (Rieske^red^). TPA binding (**2**) displaces a water molecule and primes the enzyme for a reaction with O_2_. The positions of residues involved in anchoring the substrate (R390, R309) are indicated. O_2_ binds in an end-on fashion to form (**3a**) the ferric-superoxy intermediate. One electron donation from the Rieske cluster forms a peroxy adduct, which can either heterolytically cleave to yield a high valent iron-oxo species (**3b**) or release H_2_O_2_ following diprotonation in the unproductive, uncoupling pathway (labeled *uncoupled*). A species resembling (**3a**) or (**3b**) (shown together in the dashed box) may react with TPA to form (**4**) an epoxide and ferric-hydroxy intermediate. The ferric-OH attacks the epoxide, which opens and protonates to form the product. Rereduction and hydration of the active site occur as the product departs, returning the active site to its starting state. The *cis*-*1S,2R*-DCD isomer is drawn as the presumptive product based on the observed products of the reactions between NADH/O_2_ and 2-NH_2_-TPA or 2-OH-TPA. (*B*) Hydroxylation of the ring 1,2-carbons of 2-NH_2_-TPA yields an intermediate analogous to DCD. Arrows show the pathway for rearomatization and loss of CO_2_ + NH_3_.

Many ROs are known either to accept multiple substrates (32) or to uncouple reductive O_2_ activation from substrate oxygenation, expending NAD(P)H without oxygenating the organic substrate and releasing either H_2_O_2_ or water. The substrate or analog binds near to the mononuclear Fe(II), displacing water and opening a coordination position where O_2_ can be reductively activated. The Fe/O_2_ species can either productively oxygenate the substrate or when an uncoupler is bound, break down to yield water or H_2_O_2_ (29, 40, 41) (Scheme 1 in *Discussion*).

**Fig. 3.**
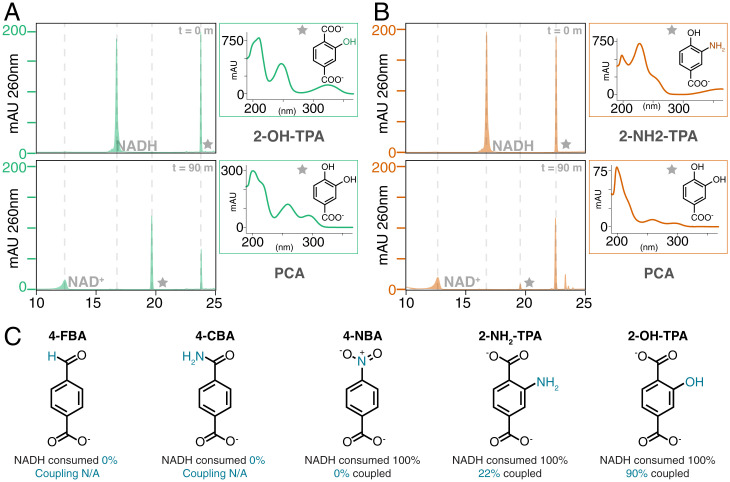
TPADO permits only structurally conservative substitutions on TPA analogs. Consumption of NADH and substrate analog following incubation in air with the TPADO/reductase system was measured via HPLC. (*A* and *B*) HPLC chromatograms, in milliabsorbance units (mAU) versus retention time, were measured at the outset (*Upper*) and 90-min end point (*Lower*) of the reaction with 2-OH-TPA (*A*) and 2-NH_2_-TPA (*B*). Stars indicate the peaks correlating to the adjacent UV/vis callout. Retention time and UV/vis spectra of the products for both the 2-OH-TPA and 2-NH_2_-TPA reactions match those of PCA, although the latter achieved substantially lower coupling. The observed PCA product for both 2-substituted TPA analogs definitively indicates 1,2-dioxygenation. (*C*) Analogs 4-FBA and 4-CBA showed no NADH consumption, while 4-NBA showed 100% consumption of NADH with no aromatic product formation (0% coupled). 2-NH_2_-TPA and 2-OH-TPA showed 22 and 100% coupling, respectively.

The specificity of TPADO was assessed by analyzing substrate consumption at a fixed time point (90 min) after incubating TPADO/reductase with stoichiometrically limiting NADH and either TPA (Fig. 2*A*) or a structural analog (Fig. 3 and *SI Appendix*, Figs. S12–S14). The stoichiometry of the aromatic substrate as a function of NADH usage was measured by integrating their respective HPLC peaks using a no substrate/no analog control sample as a baseline. Of a series of compounds screened, only TPA and closely related derivatives with hydroxyl and amino substituents at the ring C2 position were oxidized (Fig. 3). Percentage coupling was calculated as (moles substrate consumed) (moles NADH consumed above baseline)^−1^ × 100%. Both TPA and 2-hydroxy terephthalate (2-OH-TPA) exhibited 100% coupling, while 2-amino terephthalate (2-NH_2_-TPA) showed 22% coupling (Fig. 3).

Analogs with conservative substitutions to one of the carboxylates (4-nitrobenzoic acid [4-NBA], 4-carbamoylbenzoic acid [4-CBA], and 4-formylbenzoic acid [4-FBA]) yielded no detectable oxygenated products (Fig. 3*C*). 4-FBA and 4-CBA did not stimulate NADH oxidation above baseline (*SI Appendix*, Figs. S12 and S13), while 4-NBA acted as an efficient uncoupler, promoting full consumption of NADH without reduction in the substrate analog signal (*SI Appendix*, Fig. S14). Taken together, these results demonstrate an extraordinarily high level of fidelity for TPA and very closely related diacids as principal substrates of the enzyme.

### TPADO Converts 2-OH-TPA and 2-NH_2_-TPA to PCA.

TPA analogs with -NH_2_ or -OH substituents at the ring 2 position were converted to a product having a retention time and UV/vis absorbance spectrum identical to those obtained for a PCA analytical standard (CAS Registry number 99-50-3) (Fig. 3 *A* and *B* and *SI Appendix* Fig. S10 and Table S1). 1,2-*Bis*-hydroxylation uniquely leads to an intermediate where decarboxylation and ring rearomatization can proceed in conjunction with protonation and spontaneous loss of the ring 2 substituent (*Discussion*). The observation of PCA as the product in both cases, therefore, indicates that hydroxylation must take place at the 1,2-ring (and not, for example, the 1,6-ring) carbons.

### TPA Dioxygenase Has a Canonical α_3_β_3_-Structure.

**Fig. 4.**
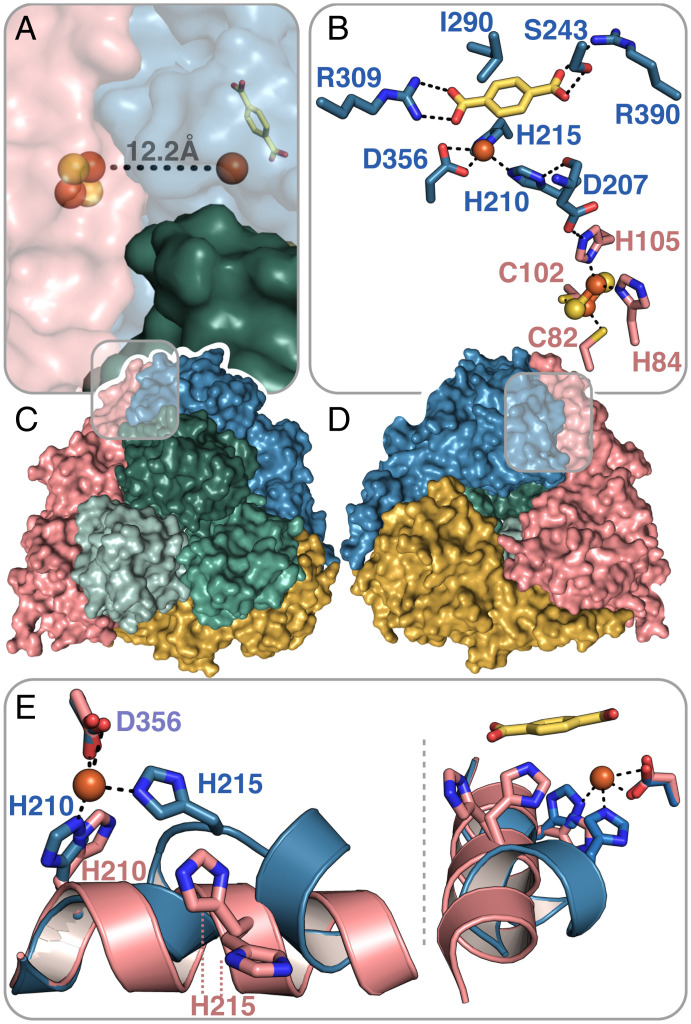
TPADO structure shows TPA binding interactions. (*A* and *C*) *Top* view of the α_3_β_3_-heterohexamer. The β-subunits are colored in shades of green, and the α-subunits are colored in pink, yellow, and blue. An iron–sulfur cluster and mononuclear iron cofactors are shown as spheres, exhibiting a head-to-tail intersubunit pathway for electron transfer. (*B* and *D*) The *bottom* view shows the trimeric architecture of the α-subunits. The bound TPA substrate is shown in yellow for the blue-colored α-subunit. Salt bridges with R309 and R390 and a hydrogen-bonding interaction with S243 are shown. The ferrous ion, which is coordinated by the side chains of residues H210, H215, and D356, is connected to the Rieske-type [2Fe-2S] cluster of a neighboring α-subunit (pink). (*E*) Superposition of two α-subunits of the TPA-bound structure shows two different conformations of an α-helix within the active site, which contains the ferrous ion-coordinating histidines H210 and H215. The pink α-helix is stabilized by crystal contacts, whereas the blue α-helix is kinked to allow the coordination of the ferrous ion by H215 when bound to TPA.

To understand the structural arbiters of substrate recognition, we determined the crystal structures of TPADO in the ligand-free state to 2.28 Å resolution and ligand-bound structures with TPA to 2.08-Å resolution and 2-OH-TPA to 1.95 Å resolution. During model building and refinement, clear continuous protein density was observed outside the α_3_β_3_-domains and identified as a single lysozyme molecule (*SI Appendix*, Fig. S15). In a rather fortuitous crystal packing, the lysozyme protein effectively acts as a crystallization chaperone that “glues” the TPADO molecules together (*SI Appendix*, Fig. S16), a function that, to our knowledge, has not been previously reported for lysozyme. More specifically, one lysozyme molecule forms interactions to five TPADO molecules and binds to both α- and β-subunits. TPADO forms an α_3_β_3_-heterohexamer, in which three catalytic α-subunits form a trimeric head-to-tail assembly atop a triad of noncatalytic β-subunits (Fig. 4*A*). Like other ROs, a Rieske domain containing a [2Fe-2S] cluster and a catalytic domain that comprises the TPA substrate and ferrous ion binding site (Fig. 4 *B* and *C*) were located in each α-subunit (25). Residues H210, H215, and D356 coordinate the ferrous ion in the active site, which is 12.2 Å away from the [2Fe-2S] cluster of a neighboring α-subunit (Fig. 4*A*) and connected via the amino acid side chains H210, D207, and H105 (Fig. 4 *B–D*). The [2Fe-2S] cluster within the same α-subunit is, by contrast, 42 Å away, where it contributes to the neighboring reaction site.

The mononuclear Fe-coordinating residues H210 and H215 are located on an α-helix spanning residues 208 to 220. In the substrate-free structure, H215 does not coordinate the ferrous ion, and in all three α-subunits, the helix conformation is the same. From residue H215 onward, this helix is disordered in two of the three α-subunits, while the third is stabilized by crystal contacts with a symmetry-related molecule (*SI Appendix*, Fig. S17 *A* and *B*). After crystal soaking with substrates TPA or 2-OH-TPA, the formerly disordered helix residues are ordered but partially unfolded, thereby allowing H215 to coordinate the ferrous ion (Fig. 4*E* and *SI Appendix*, Fig. S17 *C* and *D*). This suggests altered dynamics of the system upon substrate binding. The helix stabilized by crystal contacts in the apo structure remained in its orientation upon substrate binding and did not unfold to coordinate the iron.

TPA binding is supported by multiple ionic, hydrogen-bonding, and hydrophobic interactions. The substrate was located next to the mononuclear iron in an orientation confirmed by clear unbiased *F*_o_–*F*_c_ difference electron density (*SI Appendix*, Fig. S18). One of the carboxylate groups from the bound TPA forms a salt bridge with R309, positioning the adjacent ring carbons 3.9 to 4.1 Å away from the reactive iron in an orientation that could promote reaction with an activated Fe/O_2_ species. The side chain of I290 forms a hydrophobic π-interaction with the aromatic ring of TPA, while the second carboxylate group forms a hydrogen bond with S243. Additionally, a potential salt bridge between R390 and this carboxylate is observed, but the distance varies among the α-subunits between 2.9 and 4.6 Å. This apparent flexibility in the R390 side chain is reflected in its high B factors and could have functional significance. For example, residue R390, as well as the α-helix mentioned above and subsequent residues, forms the opening of a potential site for TPA entry or product release that may have a gate function (*SI Appendix*, Fig. S19). The TPA binding pocket is more open within the substrate-free structure because the α-helical residues from H215 onward are disordered and not part of the model; in total, residues 215 to 227 are missing.

### TPA Exhibits a Unique Binding Mode.

**Fig. 5.**
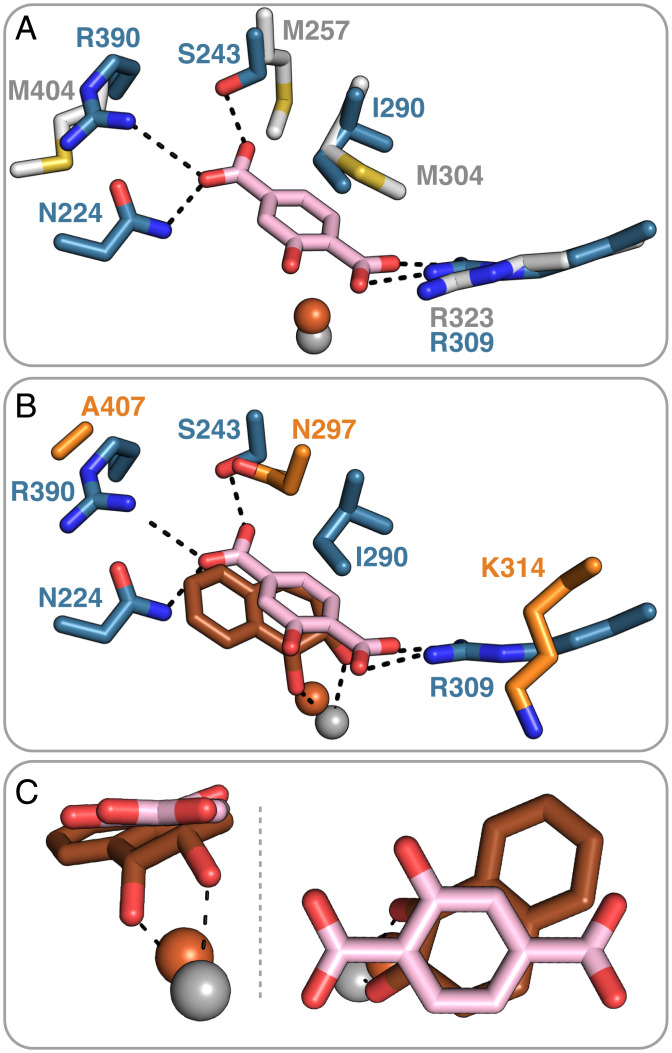
Active site structure of TPADO with 2-OH-TPA bound is shown superimposed on the structure of (*A*) NagGH and (*B* and *C*) product-bound NDO. (*A*) Superposition of the blue-colored TPADO α-subunit with the α-subunit of its closest structural homolog NagGH (gray), which has no substrate bound (PDB ID code 7C8Z), highlights the unique roles played by S243 and R390 in accommodating the substrate (38). (*B*) Superposition with the α-subunit of NDO (orange) with its dioxygenated product bound (PDB ID code 1O7P) (30). (*C*) Closer views of the overlay of the NDO product (orange) with 2-OH-TPA substrate and their relative positions to the ferrous ion. Overlap of the 1,2-diol portion of the NDO product with the ring 1,2-carbons of 2-OH-TPA suggests that these are the sites of hydroxylation.

A search in the current structural databases utilizing the Dali protein comparison server (42), highlighted NagGH (36, 43) as the closest structural homolog to TPADO, with rmsd values of 1.5 and 1.3 Å and sequence identities of 42 and 26% for the α- and β-subunits, respectively. Furthermore, the α-subunits of related toluene 2,3-dioxygenases, biphenyl dioxygenases, and NDOs are significantly more divergent, with rmsd values ranging between 2.4 and 2.8 Å. A superposition of TPADO with NagGH illustrates their similar quaternary structures (*SI Appendix*, Fig. S20). While no substrate is present in the NagGH structure for comparison, it appears unlikely that NagGH could bind TPA because the side chain of M257, which replaces the S243-TPA hydrogen-bonding interaction observed in TPADO, would interfere with the carboxylate group of TPA (Fig. 5*A*).

The salt bridge involving one of the TPA carboxylate groups is conserved in both TPADO (R309) and the NagGH (R323) from *Ralstonia* sp. (Fig. 5*A*) (38), although an equivalent H bond to R390 on the opposite end of the substrate is absent. Several additional Rieske dioxygenases possess a positively charged residue (R or K) at the same position as R309 in a pocket that is otherwise largely hydrophobic, but in those cases, the homologous side chain points away from the substrate, as observed in the NDO structure (Fig. 5*B*) (44). Moreover, the residues involved in electron transfer from the Rieske cluster to the mononuclear iron superimpose well between NagGH and NDO (*SI Appendix*, Fig. S20).

The structure of TPADO bound to 2-OH-TPA is close to identical to the complex with TPA, but surprisingly, the electron density for this ligand strongly suggests that the 2-hydroxy group is oriented toward the hydrophobic part of the active site and not toward the polar part, where hydrogen-bonding interactions with the carbonyl oxygen of V205 would appear possible (*SI Appendix*, Fig. S21). It is possible that this binding orientation of 2-OH-TPA promotes catalysis by substrate destabilization, that it favors product release, or that it represents a nonproductive binding mode. To investigate the latter possibility, we aligned the 2-OH-TPA–bound structure with a product-bound NDO structure (Protein Data Bank [PDB] ID code 1O7P) (30). From this alignment (Fig. 5 *B* and *C*), the *cis*-dioxygenated carbons of the (*1R,2S*)-*cis*-1,2-dihydroxy-1,2-dihydronaphthalene product appear to align with the 1- and 2-carbons of 2-OH-TPA. These carbons are consequently implicated as the sites of hydroxylation based on the observed formation of PCA as the ultimate product, suggesting that 2-OH-TPA occupies a productive binding mode. This also suggests, by analogy, that the stereochemistry of product of the TPA reaction is predicted to be *cis*-*1S,2R*-DCD.

### Sequence Similarity Network Analyses Relate the TPADO α-Subunit to a Subgroup of ROs That Hydroxylate Hydrophilic Monoaryls.

Classic RO subtyping by Kweon et al. (33) focused on the different domain organizations that mediate electron flow between the reductase and RO active site. A subsequent structure-based sequence alignment of 121 then available catalytic α-subunits was carried out by Capyk and Eltis (34). This analysis resulted in a first-ever phylogenomic map and suggested how gene fusion events gave rise to the Rieske/mononuclear Fe α-subunit.

**Fig. 6.**
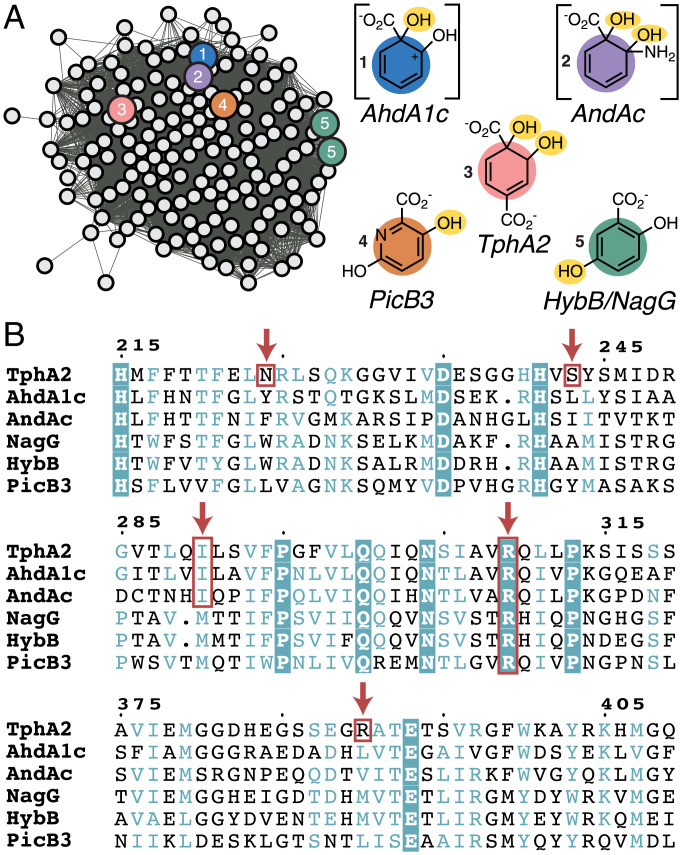
Sequence relationships of aryl carboxylate ROs. (*A*) A single cluster from a full SSN generated for PFAM00848 (*SI Appendix*, Fig. S22) containing 1,812 RO α-subunit sequences, including TphA2 (α-TPADO) and others with characterized functions relating to aryl carboxylate oxygenation (indicated by circles with color), is shown. To the right of the cluster are structures illustrating where the enzymes are proposed to hydroxylate their aryl carboxylate substrates (O_2_-derived hydroxyl groups are highlighted in yellow). Intermediates are shown in brackets to clarify where hydroxyls are installed prior to their spontaneous breakdown to yield CO_2_, H_2_O, and catechol (AhdA1c) or CO_2_, NH_3_ (protonating to form NH_4_^+^ at neutral pH), and catechol (AndAc). (*B*) A multiple sequence alignment of the five functionally characterized homologs from *A*. Orange boxes and arrows indicate residues proposed to interact with a substrate. R309 is highly conserved in these five sequences and across the cluster, suggesting that it has a functionally significant role. N224, S243, and R390 are only found in TPADO. I290 is conserved in TphA2, AhdA1c, and AndAc.

Just over 45,000 α-subunit homologs are currently known, permitting subtyping of a much broader scope. Their sequences, members of protein family (Pfam) PF00848, were submitted to an “all-by-all” comparison via the Enzyme Function Initiative’s network analysis algorithm (Fig. 6 and *SI Appendix*, Fig. S22) (45). Consistent with earlier findings from Capyk and Eltis (34), we observed that a single subgroup of the massive and diverse family has been experimentally oversampled in prior work (cluster 2) (*SI Appendix*, Fig. S22). This node contains several aromatic/polyaromatic hydrocarbon hydroxylases of interest for bioremediation purposes, including NDO and biphenyl dioxygenase (46).

Beyond this large subgroup, ROs with similar substrate and/or reaction types, where known, were grouped together in this analysis, suggestive of sequence-based family subdivision along functional lines (*SI Appendix*, Fig. S22). The cluster of sequences to which the α-subunit of TPADO (TphA2) belongs (Fig. 6) also contains NagG, the α-subunit of salicylate 5-hydroxylase, consistent with identification of the latter via the DALI search for TPADO structural relatives. Additionally, other functionally annotated members of the TPADO subfamily were all associated with aryl carboxylate substrates [anthranilate (36), picolinate (47), and salicylate] like TPA, although TPA is the only dicarboxylate of the group.

A sequence alignment of the α-subunits of these annotated members of the TphA2 sequence cluster showed that, of all the residues making direct contact with the substrate in the TPADO structure, only R309 is conserved. Key portions of this alignment are shown in Fig. 6*B* and *SI Appendix*, Fig. S23 for TPA-consuming organisms including *I. sakaiensis*, where the degree of conservation is especially high. As highlighted in Fig. 5*A*, the analogous arginine in NagG (R323) is proposed to form a salt bridge with the lone carboxylate of the substrate, positioning it for monooxygenation at the ring 5-carbon [yielding gentisate (48)]. This active site arginine is also conserved in the α-subunits of hydroxypicolinate 3-monooxygenase (47), AndAc (36), and AhdA1c (catechol product) (49, 50). A similar role in positioning the substrate via the carboxylate could be proposed for the conserved arginine in the other enzymes, although the sites of ring mono- or dioxygenation vary (Fig. 6*A*). In TPADO, N224, S243, and R390 interact with the carboxylate at the nonreactive end of TPA but are not conserved across the subfamily. This observation suggests that they may be important for productively positioning dicarboxylate substrates.

## Discussion

Plastic bioconversion using microbial enzymes depends on understanding and ultimately, improving the properties of the responsible enzymes. Hydrolyzable ester linkages are ubiquitous in biology, and aromatic compounds bearing polar substituents are abundant in the metabolic pathways of diverse organisms. The presence of both ester linkages and an aromatic building block with a polar substituent in PET is consistent with this plastic constituting a readily available carbon source for bacterial consumption in the biosphere.

TPADO is an α_3_β_3_-nonheme RO, which catalyzes the NADH-dependent dioxygenation of TPA, the aromatic subunit of PET. Like the better-studied cytochrome P450s, ROs catalyze a wide range of oxidations and oxygenations of diverse substrates, increasing their water solubility and activating them for further metabolism (51). However, unlike cytochromes P450, certain members of the RO family are capable, perhaps uniquely, of catalyzing the *cis*-dihydroxylation of an aryl ring. The reaction depends on the proper positioning of the substrate relative to an activated O_2_ species that forms at a mononuclear iron center, where both O atoms are poised to react on the same side of the plane defined by the substrate’s aromatic ring (32). Dihydroxylation results in both the loss of aromaticity and the formation of two new chiral centers in the product, DCD. This product, in turn, is well situated for further catabolism via an exergonic step, in which CO_2_ is produced, NADH is regenerated, and the ring is rearomatized to yield a valuable and versatile metabolite, PCA (52). This elegant metabolic arrangement, in which reductant is intrinsically recycled within the pathway, suggests that, despite the complexity of the TPADO/reductase system and ROs in general, this system offers a compelling starting point for engineering an efficient route for TPA bioconversion to PCA.

PET is an abundant, xenobiotic source of environmental TPA, suggesting that TPA might not be the primary but perhaps, a secondary substrate of TPADO. We noted that the bacterial TPADO studied here was nonetheless highly efficient and extraordinarily specific for TPA (*K*_M_ = 9.6 μM, *k*_cat_/*K*_M_[TPA] = 2.1 M^−1^ s^−1^), coupling NADH oxidation to TPA dihydroxylation with 100% fidelity and excluding a variety of structurally related compounds as potential substrates (Fig. 3). This observation is consistent with an early report on the *Comamonas* E6 TPADO indicating that neither the *iso*- nor the *ortho*-benzene dicarboxylate regioisomers of TPA (*para*-benzene dicarboxylate) were substrates for the enzyme (17). Two major exceptions identified here are 2-OH-TPA and to a lesser extent, 2-NH_2_-TPA, where TPADO catalyzed the conversion of each to PCA.

Each of the benzene dicarboxylate regioisomers is used in the production of plastics, whether as a repeating subunit in PET, as a synthetic precursor for monomers used in plastics, or as a noncovalently bound plasticizer (51). The *iso*- and *ortho*-benzene dicarboxylates (phthalates) serve one or both of the latter functions in several plastics. *Ortho*-phthalate has been of special concern as an endocrine disruptor with the potential to leach out of consumer products and into water (53). Bacteria that can degrade each of these compounds have been identified (54), indicating that enzymatic adaptation to these plastic-relevant compounds is not unique to one or a few strains. Recent work even suggests possible biogenic sources for and derivatives of phthalates (55), which could have helped drive RO diversification.

The *Comamonas* E6 strain has served as a paradigmatic plastic monomer degrader, capable of using each of the benzene dicarboxylate regioisomers as a sole source of carbon and energy (54), via separate operons encoding three distinct RO enzymes. While the TPADO has an α_3_β_3_-subunit structure, the *ortho*-phthalate dioxygenase (PDO) from *Comamonas* E6 displays an α_3_α_3_-hexameric structure. It is possible that *iso*-PDO and *ortho*-PDOs from related strains have similar architecture. The strain E6 *iso-*/*ortho*-PDO α-subunits share 32% identity with one another, forming a separate lineage from the α-subunits of TPADO and other α_3_β_3_-ROs with which they share ≤19% identity (56). Accordingly, the α-subunits of TPADO and a recently reported *ortho*-PDO structure superimpose poorly (*C. testosteroni* KF1 PDO) (*SI Appendix*, Fig. S24) (54). The active site of this PDO exhibited ionic/hydrogen-bonding interactions between the bound *ortho*-phthalate carboxylates and an Arg-Arg-Ser triad that is unconserved in the TPADO sequence or tertiary structure. Hydrophobic interactions were observed surrounding the benzylic ring. TPA was able to bind with some observed hydroxylation in the same PDO pocket, although with 80% uncoupling and a much lower *k*_cat_/*K*_M_ than for *ortho*-phthalate. These results suggest that TPADO and PDO are each well adapted to their preferred benzene dicarboxylate regioisomer.

A large-scale network analysis of >45,000 sequences of RO catalytic α-subunits was generated, aimed at understanding the origins of TPA bioconversion. Consistent with the unique homohexameric structures identified by Capyk and Eltis (34), PDO α-subunits from *Comamonas* are sufficiently sequence distant that they are not grouped with the same PFAM family (PF00848) and are not found in this sequence similarity network (SSN). Analysis of the network identified a subcluster containing the closest sequence relatives to TPADO (Fig. 6 and *SI Appendix*, Fig. S24). While only a few members of the subcluster are characterized in the literature, they are all known to catalyze reactions with aryl-carboxylic acids, which structurally resemble TPA. Many of the nodes in the cluster shown in Fig. 6*A* are annotated as similar to AhdA1c, the catalytic subunit of salicylate-1-monooxyenase (IPR043264). A multiple sequence alignment revealed a cluster-wide conserved arginine, which forms a salt bridge to one of the carboxylate groups of TPA (R309) (Fig. 4) and which is proposed to engage in a similar interaction with salicylate based on structural characterization of NagGH (38). An additional set of residues (N224, S243, and R390) has side chains within hydrogen-bonding distance of the second carboxylate in the TPA and 2-OH-TPA costructures with TPADO (Figs. 4 and 5) described here. These are not conserved in the other functionally annotated sequences highlighted in Fig. 6. This conserved motif may, therefore, offer a means of identifying diverse TPADOs from sequence databases. Additional interesting findings resulting from the SSN analysis cannot be discussed here at length. We are supplying the network file for readers to explore the vast sequence space. Many of the clusters in the SSN are made up entirely of sequences with no known function, suggesting that a plethora of catalytic diversity remains to be discovered.

A structure-based mechanism can be proposed to explain the observed products of the TPADO-catalyzed reactions with TPA, 2-OH-TPA, and 2-NH_2_-TPA considering the data presented here and two additional observations. First, the network analysis revealed a close sequence relationship between the α-subunits of TPADO and anthranilate dioxygenase from *Burkholderia cepacia* DBO1 (36). This organism can grow with anthranilic acid as a sole carbon source, where it is proposed that its RO functions to dihydroxylate anthranilate, which then spontaneously deaminates and decarboxylates to yield PCA (36). Second, extensive prior work has shown that NDO, perhaps the best-studied RO, can catalyze a variety of oxidations depending on the position of the substrate in the wild type (WT )and variant enzymes relative to the site of O_2_ activation. Aryl carbons that are closest to the mononuclear iron (30, 57) are generally prioritized for hydroxylation.

A mechanism (Scheme 1*A*), taking these observations and prior work with ROs into account, would proceed as follows. Substrate binding near to the mononuclear Fe in fully reduced TPADO is expected to displace an iron-bound water molecule from TPADO (29, 40, 41). Here, we observed an unexpectedly large change in the conformation of a helix containing two Fe-ligating histidine residues in response to substrate binding (Fig. 4*C*), consistent with the role of the substrate in permitting binding and reductive activation of O_2_. The initially formed ferric-η^1^-superoxy intermediate (48) or the related Fe-oxo/Fe-hydroxo species could in principle be the oxygenating species. This species would attack the nearest available substrate carbon (Fe ring C2 = 3.6 Å). Addition of a second active site electron from the Rieske cluster yields the ring 1,2-epoxide, adjacent to the mononuclear ferric-hydroxyl. Nucleophilic attack of the hydroxyl at ring C1 forms the hydrogenated, dearomatized, *cis*-diol product.

Dioxygenation of the ring 1,2-carbons of 2-OH-TPA by this route would yield an unstable gem-diol at the ring C2 position. This is expected to readily dehydrate and decarboxylate to yield PCA and CO_2_ in the presence of an aqueous proton source to react with the hydroxyl-leaving group. An analogous mechanism has been postulated for the close sequence relative of TPADO, anthranilate dioxygenase, in which the substrate, a 2-amino benzoate, is initially dihydroxylated and hydrogenated at the ring 1,2-carbons. Protonation of the C2-NH_2_ group would catalyze the breakdown of the product to yield catechol, CO_2_, and ammonia, which would rapidly acquire a proton to form ammonium cation under neutral, aqueous conditions. The dicarboxylate analog of anthranilate (2-NH_2_-TPA) serves as a substrate of TPADO with the corresponding deaminated product PCA, although the poorer amino-leaving group leads to a lower level of productive turnover compared with 2-OH-TPA. The close relationship between TPADO and anthranilate dioxygenase and the proximity of the reactive Fe(II) to the 1,2-carbons of TPA or 2-OH-TPA in the TPADO structures presented here (Fig. 5*C*) suggest an analogous route to DCD or PCA production in TPADO (Scheme 1*B*).

Together, these observations connect the experimentally determined binding interactions between TPADO and its substrates to an RO sequence subtype and a potential sequence motif specific for *para*-aryl-dicarboxylate substrates like TPA. These, in turn, provide strong support for a proposed pathway for the TPADO-catalyzed reaction that can now be optimized for future applied work.

## Materials and Methods

TPADO and the reductase were heterologously expressed in *E. coli* and isolated in high yields via nickel affinity chromatography. Enzyme activity was monitored continuously via UV/vis monitoring of NADH disappearance and discontinuously via separation of reaction components via HPLC. Identities of starting material and products were detected with both mass spectrometric and diode array UV/vis detectors. Quantification of reaction materials was carried out by integrating peak areas from chromatograms recorded at distinct absorption maxima. TPADO was crystallized in the substrate-free state and soaked with substrates TPA and 2-OH-TPA overnight. The crystal structures were solved by molecular replacement using structural homologs for the α- and β-subunits. Coordinates for the resulting apo and substrate-bound structures have been deposited in PDB (ID codes 7Q04, 7Q05, and 7Q06). Structural superpositions of TPADO with NagG (7C8Z) and NDO (1O7P) were generated and visualized in PyMol. An SSN of the family of proteins that the catalytic domain of TPADO belongs to (PF00848) was generated with EFI-EST web tools and visualized in Cytoscape. Detailed methods are provided in *SI Appendix*.

## Supplementary Material

Supplementary File

## Data Availability

Crystallographic coordinates have been deposited in PDB (ID codes 7Q04, 7Q05, and 7Q06) (58). All additional raw sequence and spectroscopic data used in this paper are available in the *SI Appendix*.
