# Supplementary material for: Biochemical and structural characterization of an aromatic ring–hydroxylating dioxygenase for terephthalic acid catabolism
Source: Proc Natl Acad Sci U S A. 2022 Mar 21;119(13):e2121426119. doi: 10.1073/pnas.2121426119 (PMC9060491; doi:10.1073/pnas.2121426119)
Supplement: Supplementary File [file pnas.2121426119.sapp.pdf]

Supplementary Appendix for

Biochemical and structural characterization of an aromatic ring-hydroxylating dioxygenase for terephthalic acid catabolism

William M. Kincannon,[1] Michael Zahn,[2,4] Rita Clare,[1,4] Ari Romberg,[1] James Larson,[1,] Jessica Lusty-Beech,[1,4,] Brian Bothner,[1] Gregg T. Beckham,[3,4] John E. McGeehan,[2,4,*] Jennifer L. DuBois,[1,4,*]

1. Department of Biochemistry, Montana State University, Bozeman MT, United States
2. Centre for Enzyme Innovation, University of Portsmouth, Portsmouth, United Kingdom
3. Renewable Resources and Enabling Sciences Center, National Renewable Energy Laboratory, Golden CO United States
4. BOTTLE Consortium, Golden CO, United States

*Correspondence: john.mcgeehan@port.ac.uk; jennifer.dubois1@montana.edu

Classification: Biological Sciences, Applied Biological Sciences

*John E. McGeehan, Jennifer L. DuBois
Email: john.mcgeehan@port.ac.uk; jennifer.dubois1@montana.edu

This PDF file includes:

Supplementary methods
Figures S1 to S24
Tables S1 to S4
SI References

Detailed Experimental Methods.

Generation of expression constructs

Genes encoding the TPA oxygenase alpha subunit (*tphA2*, BAE47077), beta subunit (*tphA3*, BAE47078) and reductase (*tphA1,* BAE47080) were synthesized in an IPTG-regulated pET expression vectors by Genscript. For TPADO, a TEV-cleavable C-terminal His_6 tag was appended to the α-subunit (TphA2), while the β-subunit (TphA3) was untagged**.** The two polypeptides were co-expressed in the same parent pET-DUET vector. For the reductase, a C-terminal His_6 tag was appended using pET45b. Vector maps and complete protein and gene sequences are given in Fig S1-S2.

Overexpression of recombinant dioxygenase

Lemo21(DE3) *E. coli* cells (New England Biolabs) harboring the pETDuet-TPADO construct were grown overnight at 37 °C in 100 mL LB starter media (5 g sodium chloride, 5 g yeast extract, 10 g tryptone per 1 L deionized water). The next morning, 500 mL TB media (1) in 2.8 L Fernbach flasks was seeded with 1/100 volume of overnight starter culture. The media was also supplemented with ampicillin (0.1 mg/mL), chloramphenicol (0.034 mg/mL), and rhamnose (2 mM). Cultures were incubated at 37 °C with agitation at 225 rpm until the OD_{600nm} reached ~4 (after ~5-6 h). Then, the flasks were cooled by complete submersion in ice for 45 minutes. After this time, the ice-chilled cultures were supplemented with 0.1 mM ferric chloride and 10 mg/L L-Cysteine hydrochloride monohydrate. Finally, protein production was induced with 1 mM IPTG and the culture flasks returned to incubation at 25 °C with agitation at 200 rpm. 18 h after the induction, cells were harvested 18 via centrifugation at 4 °C. The cell paste was frozen with liquid nitrogen, and stored at -80 °C until needed.

Purification of recombinant dioxygenase

Cell pellet was resuspended in buffer A (20 mM TrisHCl, 150 mM NaCl, 10% glycerol w/v%, pH 8.0), with approximately 20 g cell paste to 160 mL of buffer. The protease inhibitor phenylmethylsufonyl fluoride (PMSF) and chicken egg white lysozyme were added to 1 mM and 1 mg/mL, respectively. The solution was incubated at room temperature, with stirring, for 15 minutes. Then the lysate solution was sonified with a Branson digital sonifier, with stirring while incubated in an ice bath. The solution was sonified for 20 minutes, with a pulse sequence of 10 seconds on and 30 seconds off, at 50% amplitude. The sonified lysate was then clarified via centrifugation (18,000 x g, 30 minutes, 4 °C).

Clarified lysate was loaded onto 3 x 5 mL HisTrap (Cytiva) nickel affinity columns that were pre-equilibrated in buffer A. The column resin was then washed with 10 column volumes of buffer A, and then a second wash step with 60% buffer A and 40% buffer B (same as A, except with 0.25 M imidazole) for 10 column volumes. Finally, resin-bound protein was eluted via a step to 100% buffer B. Fractions (3 mL) were collected throughout the loading, washing, and eluting steps of the procedure, and analyzed for TPADO presence by SDS-PAGE. Significantly pure protein found in the final elution was concentrated to 4 mL with 30 MWCO amicon centrifugal filtration devices, and desalted into buffer A using a DG-10 column (BioRad). The protein was concentrated to 2 mL, dispensed into 0.05 mL aliquots, flash frozen with liquid nitrogen, and stored at -80 °C until needed.

Protein for crystallography was purified from 1 L scaled TB cultures, instead of 500 mL. Protein for crystallography was also buffer exchanged into Buffer A with an additional 2 mM dithiothreitol by dialysis with a 3 MWCO membrane, at the final steps of the purification. Buffer was changed two times over 24 h, and the protein was then concentrated and flash frozen as described above.

Overexpression of recombinant reductase

Lemo21(DE3) *E. coli* cells (New England Biolabs) harboring the pET-DO-Red construct (Fig S1) were grown overnight at 37 °C in 100 mL LB starter media (5 g sodium chloride, 5 g yeast extract, 10 g tryptone per 1 L deionized water). The next morning, 500 mL of TB media (1) in a 2.8 L Fernbach flask was seeded with 1/100 volume of overnight starter culture. The media was also supplemented with ampicillin (0.1 mg/mL), chloramphenicol (0.034 mg/mL), and rhamnose (2 mM). The seeded medium was then incubated at 37 °C with agitation at 225 rpm until the OD_{600nm} reached ~2.5 (or ~5 hours). Then, the culture was cooled by complete submersion in ice for 45 minutes. After this time, the ice-chilled culture was supplemented with 0.1 mM ferric chloride, 10 mg/L L-Cysteine hydrochloride monohydrate, and 0.2 mg/mL (−)-Riboflavin. Finally, protein production by the cells was induced with 0.4 mM IPTG, and cultures were returned to incubation at 20 °C with agitation at 200 rpm. The cells were harvested 18 hours after induction via centrifugation at 4 °C. The cell paste was frozen with liquid nitrogen and stored at -80 °C until needed.

Purification of recombinant reductase

All purification steps were conducted under anaerobic conditions, in a Coy anaerobic chamber containing 2.5% H_2/97.5%N_2 atmosphere. Cell pellet was resuspended in buffer C (20 mM TrisHCl, 300 mM NaCl, 15% glycerol w/v%, pH 8.0), with approximately 20 g cell paste to 160 mL of buffer. PMSF and chicken egg white lysozyme were added to 1 mM and 1 mg/mL, respectively. The solution was incubated at room temperature with stirring for 15 minutes. Then the lysate solution was sonified with a Branson digital sonifier, with stirring while incubated in an ice bath. The solution was sonified

for 20 minutes, with a pulse sequence of 10 second on and 30 seconds off, at 50% amplitude. The sonified lysate was then clarified via centrifugation (18,000 *x g*, 30 minutes, 4 °C). Clarified lysate was loaded onto 3 x 5 mL HisTrap (Cytiva) nickel affinity columns that were pre-equilibrated in buffer C. The column resin was then washed with 10 column volumes of buffer C. Protein was eluted via a step to 100% buffer D (same as buffer C, except with 100 mM imidazole). Fractions (3 mL) were collected throughout the loading, washing, and eluting steps of the procedure, and analyzed for reductase presence by SDS-PAGE. Significantly pure protein found in the final elution was concentrated to 4 mL with 30 MWCO Amicon centrifugal filtration devices, and desalted into buffer C using a DG-10 column (BioRad). The protein was concentrated to 2 mL, aliquoted into 0.05 mL volumes, flash frozen with liquid nitrogen, and stored at -80 °C until needed.

Atomic absorption spectroscopy

Dioxygenase was analyzed for iron content with three replicate preparations. Serial dilutions of each prep were conducted to obtain 10 mL of protein samples ranging from 2 to 5 µM concentration. Then, the samples were then analyzed by flame AA spectroscopy using an Agilent 240 FS instrument (Agilent Technologies Inc.) equipped with an Fe lamp utilizing an acetylene/air mixture (11:60 psi partial pressures) as a fuel source. Concentration of iron was then determined from a 0.1–2.0 ppm standard curve created from a 1000 ppm iron AA standard solution (Ricca Chemical Company).

Steady-state assays

Initial rates of reaction under steady state conditions (low [enzyme]) were measured using a Cary 60 UV-Vis spectrophotometer under aerobic conditions. Activity assays were performed by recording absorbance changes at 340 nm upon mixing of TPADO (1.9 µM), reductase (6.0 µM), and NADH (200 µM) with terephthalate (2.5–100 µM) over time. Reactions were conducted on a 2.0 mL scale, where a buffered solution of TPADO, reductase, and NADH was reacted with terephthate in a quartz cuvette (1 cm path length, concentrations after mixing). Solutions were prepared in ambient air (~250 µM O_2) and contained 20 mM TrisHCl pH 8.0, 150 mM sodium chloride, 10% glycerol (v/v), 20% DMSO (v/v), pH 8.0 buffer at 28 °C. Rates of reactions were obtained from the slope of the initial portion of the time course using the extinction coefficient of NADH at 340 nm (6.22 mM^{-1} x cm^{-1}). Rates were measured as a function of variable TPA concentration and subsequently fit to the Michaelis-Menten hyperbolic expression.

Assays for monitoring enzyme catalyzed aromatic hydrocarbon oxygenation

Assays for HPLC analysis were conducted on a 2.0 mL scale, incubated at 28 °C, and stirred at 400 RPM with a flea stir bar. Each assay contained 20 mM TrisHCl (pH 8.0), 150 mM sodium chloride, 15% DMSO, 10 % glycerol (v/v%), 0.5 mM TPA or TPA analog, 0.35 mM NADH, 1.0 µM reductase, and 2.1 µM dioxygenase. Assays were initiated with addition of dioxygenase. After dioxygenase was added, the reaction was rapidly mixed with a pipette and incubated with stirring. Reactions were quenched with ice cold methanol to a 1:1 ratio, and centrifuged to remove debris. For the measurement of total turnover number, conditions were changed to include 20% DMSO, 3 mM NADH, 1 mM TPA, 0.3 µM reductase, and 1 µM dioxygenase.

HPLC monitoring and analysis of TPADO-catalyzed reactions

Quenched reactions were analyzed for aromatic substrate/product, NADH, and NAD^+ content using a Shimadzu HPLC instrument with a diode array detector by injecting an aliquot onto a Thermo Scientific™ Hypersil GOLD™ (4.6 mm × 250 mm, 5 µm particle size) pre-equilibrated in 100% buffer A and 0% buffer B. Buffer A consisted of 0.1% (v/v) TFA (Fisher) in water. Buffer B consisted of 0.1% (v/v) TFA (Fisher) in HPLC grade acetonitrile (Fisher). The reaction components were eluted at a rate of 1 mL/min with the following program: 0% B from 0.0 to 10.0 min, 0 to 15% B from 10.0 to 18.0 min, 15 to 75% B from 18.0 to 24.0 min, 75 to 100% B from 24.0 to 24.1 min, 100% B from 24.1 to 30.1 min, 100% to 0%B from 30.1 to 30.2 minutes, and 0% B from 30.2 to 38.0 min. Authentic TPA (Sigma-Aldrich), 2-hydroxyterephthalate (Sigma-Aldrich; 10312-55-7), 2-aminoterephthalate (Alfa Aesar; 10312-55-7), 4-nitrobenzoate (ChemImpex; 62-23-7), 4-carbamoylbenzoate (AA Blocks; 6051-43-0), and 4-formylbenzoate (AA Blocks; 619-66-9), were used to develop standard curves from integrations of peaks monitored at the wavelengths and retention times reported in Table S1. The relationship of authentic substrate concentration to peak area was used to quantify substrate content in experimental reactions. Triplicate measurements were conducted for each condition, and mean concentrations were obtained with less than 10% standard error.

Partial uncoupling of NADH consumption from substrate hydroxylation was screened for in reactions involving each of the above substrates/analogs, and observed over baseline NADH consumption in the presence of 4-nitrobenzoate. To calculate %-uncoupling, the following expression was used:

$$\{\Delta[\text{substrate}] / (\Delta[\text{NADH}]_{\text{total}} - \Delta[\text{NADH}]_{\text{baseline}})\} \times 100\%$$

Here, Δ[substrate] was determined by the diminution of the area of the substrate-associated HPLC peak in a reaction mixture, and $\Delta[NADH]_{total}$ was determined by reduction of area of the NADH-associated HPLC peak in the same reaction sample following incubation for 90 minutes. In each case, data were measured for an initial time point (t_0) immediately prior to the addition of enzymes. $\Delta[NADH]_{baseline}$ was determined in a similar fashion by measuring diminution of the area of the NADH-associated HPLC peak in a no-substrate control, before and following incubation with the TPADO/reductase enzymes.

Liquid chromatography (LC) electrospray and tandem mass spectrometry (MS-MS)

To confirm the production of DCD, a reaction containing TPA/NADH prior to addition of enzymes and a quenched TPADO/reductase-catalyzed reaction using TPA and NADH as substrates (see above) were each analyzed via reverse-phase UV-UPLC-LCMS on an Agilent 1290 infinity UHPLC equipped with DAD coupled to an Agilent 6538 high-resolution Q-TOF mass analyzer. Samples were injected onto an Acquity UPLC HSS T3 column (2.1 x 100mm, 1.8 µm particle size). Molecules were eluted at a rate of 0.2 mL/min with the following program: 1% B from 0 to 2 minutes, 1 to 15% B from 2 to 10 minutes, 15 to 75% B from 10 to 16 minutes, 75 to 99% B from 16 to 16.1 minutes, 99% B from 16.1 to 18.1 minutes, 99 to 1% B from 18.1 to 18.2 minutes, and 1% B from 18.2 to 22 minutes with A consisting of water with 0.1% (v/v) formic acid and B consisting of acetonitrile with 0.1% formic acid (v/v). The combined data from UV signal, retention time, accurate mass data, and extracted ion chromatograms were used to identify TPA, NAD^+, NADH, and DCD. These data identified ions with expected m/z for TPA and DCD, as well as several putative fragmentation products generated in the source. To confirm loss of water/carbon dioxide from DCD and TPA, the samples were re-analyzed using targeted MS-MS on TPA/DCD ions at their expected elution times by collision induced fragmentation (CID) with the source voltage reduced from 3,500 V to 3,000 V. Exact mass data are summarized in Table S3 and Figure S8.

Native protein mass spectrometry

The native assembly of the dioxygenase was analyzed with native mass spectrometry (NMS). To prevent aggregation in the absence of glycerol, TPADO (initial concentration of 40 mg/mL) was first diluted to a concentration of 1 mg/mL with 170 mM ammonium acetate (Sigma-Aldrich) pH 8. The enzyme was buffer exchanged via overnight dialysis against 170 mM ammonium acetate pH 8 using a 3.5 kDa molecular weight cut-off membrane (Thermo Fisher). Sample was introduced with in-house prepared gold-coated borosilicate glass (2) capillaries and analyzed with a Synapt G2-Si electrospray time-of-flight mass spectrometer (Waters). Analysis of 10 µM TPADO in 170 mM ammonium acetate pH 8 and 2% acetonitrile (Thermo Fisher) resulted in an unsteady signal. Reducing the concentration of ammonium acetate to 50 increased signal stability considerably. Instrument parameters were: 60 °C source temperature, trap collision energy of 10 V, and capillary voltage of 2.00 V. Data were collected in positive mode at 1 Hz scan frequency over a m/z scan range of 800-12,000.

Electron paramagnetic resonance.

Concentrated dioxygenase protein was thawed in an anaerobic chamber rapidly by holding in a gloved hand. Then, 0.3 mL of protein was treated with 0.03 mL of 0.1 M sodium dithionite (dissolved in buffer A), and mixed gently with a pipettor. The mixture was incubated at room temperature in the anaerobic chamber for 30 minutes, then transferred to an EPR tube and capped with a rubber lid to maintain anaerobicity. The tube was then removed from the chamber, and immediately frozen with liquid nitrogen. Concentrated reductase protein was thawed in an anaerobic chamber rapidly by holding in a gloved hand. Then, 0.3 mL of protein was treated with 0.03 mL of 0.1 M NADH (dissolved in buffer C), and mixed gently with a pipettor. The mixture was incubated at room temperature in the anaerobic chamber for 30 minutes, then transferred to an EPR tube and capped with a rubber lid to maintain anaerobicity. The tube was then removed from the chamber, and immediately frozen with liquid nitrogen.

X-Band CW EPR spectra of dioxygenase and reductase proteins were measured with a Bruker EMX spectrometer fitted with a ColdEdge (Sumitomo Cryogenics) 10 K waveguide in-cavity cryogen-free system with an Oxford Mercury iTC controller unit and helium Stinger recirculating unit (Sumitomo Cryogenics, ColdEdge Technologies). Helium gas flow was maintained at 100 psi. Spectral parameters were 2 mW microwave power, 100 kHz modulation frequency, and 10 G modulation amplitude. Spectra were averaged over 2 scans.

Crystallography

The Ni-affinity purified TPADO was buffer exchanged into 20 mM Tris pH 8.0, 150 mM NaCl, 10% glycerol, 2 mM DTT and concentrated to 10 mg/mL. Sitting drop crystallization trials were set up with a Mosquito crystallization robot (sptlabtech) using SWISSCI 3-lens low profile crystallization plates. Crystals grew in condition C4 (12.5% MPD, 12.5% PEG 3350, 12.5% PEG 1000, 0.3 M sodium nitrate, 0.3 M sodium phosphate dibasic, 0.3 M ammonium sulfate, 0.1 M buffer Imidazole/MES pH 6.5) of the Morpheus screen (Molecular Dimensions). Apo crystals were soaked with 4 mM TPA or 10 mM 2-OH-TPA for 24 hours before they were cryo-cooled in liquid nitrogen. Diffraction data were collected on beamline I03 at the Diamond Light Source (Didcot, UK) and automatically processed with STARANISO (3) on ISPyB. The structure was solved within CCP4 Cloud by molecular replacement with Molrep (4) using homology search models for the α- and β-subunits from

SWISS-MODEL (5) based on pdb entries 3N0Q and 3EBY, respectively. Model building was performed in Coot (6) and the structures were refined with REFMAC5 (7). MolProbity (8) was used to evaluate the final models and PyMOL (Schrödinger, LLC) for protein model visualization. Data and Refinement statistics are summarized in Table S2. The atomic coordinates have been deposited in the Protein Data Bank and are available under the accession codes 7Q04, 7Q05 and 7Q06. Search for structural protein homologs and calculation of root-mean-square deviation values were performed with the DALI server (9).

Sequence Similarity Network

The network was generated by the EFI-EST (Enzyme Function Initiative – Enzyme Similarity Tool) server from PF00848, a protein family containing putative alpha subunits for aromatic ring-hydroxylating Rieske dioxygenases. A UniRef90 was applied to the 45,076 membered Pfam to reduce the total number unique sequences in the network to 17,319. The sequence alignments were conducted with E-value of 5 and alignment score of 85 to generate the full network. A repnode network was used to condense sequences with ≥40% sequence ID into single nodes. This repnode network was colorized with the EFI-EST server, and subsequently visualized in Cytoscape v3.11.1 where a y-files organic layout was applied and singletons were manually removed, resulting in the final network shown. Only clusters with at least a single node containing a sequence of a functionally verified protein are numbered. Clusters are numbered by the "Sequence Count Cluster Number" attribute bestowed by the EFI-EST server. Table S4 lists representative UniProt IDs from each cluster that are associated with proteins/genes with published activities.

Supplementary Figures

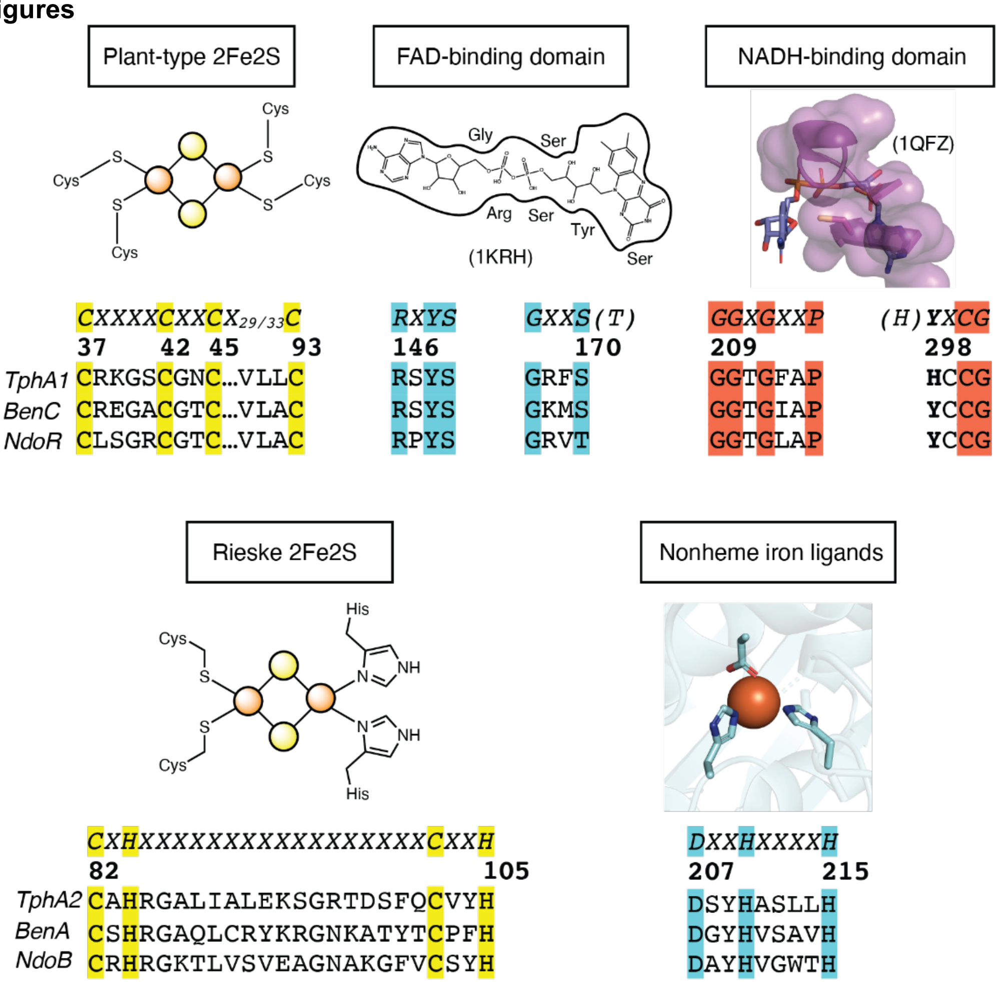

Figure S1. Conserved cofactor binding motifs in reductase and Rieske oxygenase enzymes most similar to TPADO α subunit (TphA2, bottom) and TPADO reductase (TphA1, top). Sequence alignments shown have highly conserved binding or contact residues highlighted, with a consensus sequence shown in the first line. Illustrations of how each motif is involved in cofactor binding are shown above the respective sequence alignments. BenC = benzoate dioxygenase alpha subunit; NdoB = naphthalene dioxygenase alpha subunit.

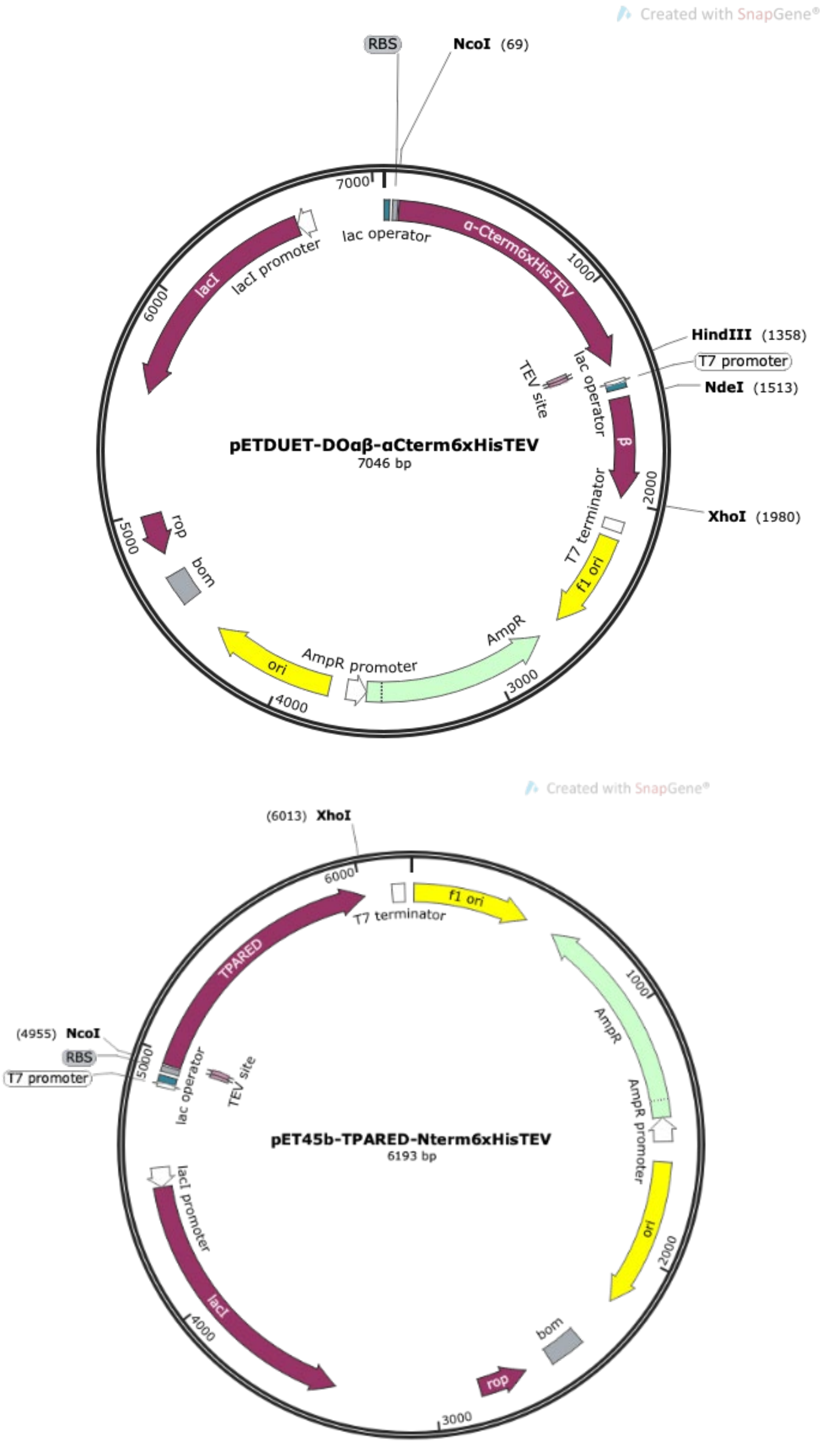

Figure S2. Plasmid maps for expressing TPADO (top) and its reductase (bottom), generated with SnapGene Viewer. A C-terminal His6 tag is appended to the α-subunit of TPADO and to the reductase.

(A) MGMQESIIQWHGATNTRVPFGIYTDTANADQEQQRIYRGEVWNYLCLESEIPGAGDFRTTFAGETPIVVVRDADQEIYAFENRCAHRGALIALEKSGRTDSFQCVYHAWSYNRQG
DLTGVAFEKGVKGQGGMPASFCKEEHGPRKLRVAVFCGLVFGSFSEDVPSIEDYLGPEICERIERVLHKPVEVIGRFTQKLPNNWKLYFENVKDSYHASLLHMFFTTFELNRLSQ
KGGVIVDESGGHHVSYSMIDRGAKDDSYKDQAIRSDNERYRLKDPSLLEGFEEFEDGVTLQILSVFPGFVLQQIQNSIAVRQLLPKSISSSELNWTYLGYADDSAEQRKVRLKQA
NLIGPAGFISMEDGAVGGFVQRGIAGAANLDAVIEMGGDHEGSSEGRATETSVRGFWKAYRKHMGQEMQA**ENLYFQG**HHHHHH*

(B) MINEIQIAAFNAAYAKTVDSDAMEQWPTFFTKDCHYRVTNVDNHAEGLAAGIVWADSQDMLTDRISALREANIYERHRYRHILGLPSIQSGDATQASASTPFMVLRIMHTGETEV
FASGEYLDKFTTIDGKLRLQERIAVCDSTVTDTLMALPL*

(C) GGGGAATTGTGAGCGGATAACAATTCCCCTCTAGAAATAATTTTGTTTAACTTTAAGAAGGAGATATACCatgggcatgcaggaaagcatcattcaatggcacggtgcgaccaac
acccgtgttccgttcggcatctataccgacaccgcgaacgcggatcaggaacagcaacgtatttaccgtggtgaggtgtggaactatctgtgcctggagagcgaaattccgggtg
cgggcgacttccgtaccacctttgcgggcgagaccccgatcgtggttgtgcgtgacgcggatcaagaaatttacgcgttcgagaaccgttgcgcgcaccgtggtgcgctgattgc
gctggaaaagagcggccgtaccgacagctttcagtgcgtttaccacgcgtggagctataaccgtcaaggtgatctgaccggcgttgcgttcgagaagggtgtgaaaggccagggt
ggcatgccggcgagcttttgcaaagaggagcacggtccgcgtaaactgcgtgttgcggtgttctgcggtctggttttcggcagctttagcgaagacgtgccgagcattgaggatt
acctgggtccggaaatctgcgaacgtattgagcgtgttctgcacaaaccggttgaagtgatcggccgtttcacccagaagctgccgaacaactggaaactgtactttgagaacgt
gaaggacagctatcacgcgagcctgctgcacatgttctttaccacctttgaactgaaccgtctgagccaaaaaggtggcgttatcgtggacgagagcggtggccaccacgttagc
tacagcatgattgatcgtggtgcgaaggacgatagctataaagaccaggcgatccgtagcgataacgaacgttaccgtctgaaggacccgagcctgctggaaggtttcgaggaat
ttgaggatggcgttaccctgcaaattctgagcgttttcccgggttttgtgctgcagcaaatccagaacagcattgcggtgcgtcaactgctgccgaaaagcatcagcagcagcga
actgaactggacctacctgggttatgcggacgatagcgcggagcagcgtaaagtgcgtctgaaacaagcgaacctgatcggtccggcgggcttcattagcatggaggatggcgcg
gttggtggctttgtgcagcgtggtattgcgggtgcggcgaacctggacgcggttattgaaatgggtggcgatcacgaaggtagcagcgagggtcgtgcgaccgagaccagcgtgc
gtggtttctggaaggcgtatcgtaaacacatgggccaggaaatgcaagcgGAGAACCTGTACTTTCAGGGTCATCACCACCACCACCACtaaAAGCTTGCGGCCGCATAATGCTT
AAGTCGAACAGAAAGTAATCGTATTGTACACGGCCGCATAATCGAAATTAATACGACTCACTATAGGGGAATTGTGAGCGGATAACAATTCCCCATCTTAGTATATTAGTTAAGT
ATAAGAAGGAGATATACATatgatcaacgagatccagattgcggcgttcaacgcggcgtacgcgaagaccgtggacagcgatgcgatggaacaatggccgaccttctttaccaaa
gactgccactatcgtgtgaccaacgttgataaccatgcggagggtctggcggcgggtattgtttgggcggacagccaggatatgctgaccgaccgtatcagcgcgctgcgtgagg
cgaacatttacgaacgtcaccgttatcgtcacatcctgggtctgccgagcattcaaagcggtgatgcgacccaagcgagcgcgagcaccccgtttatggtgctgcgtatcatgca
caccggcgagaccgaagttttcgcgagcggcgagtacctggacaagtttaccaccatcgatggtaaactgcgtctgcaagaacgtattgcggtgtgcgacagcaccgttaccgat
accctgatggcgctgccgctgtaaCTCGAGTCTGGTAAAGAAACCGCTGCTGCGAAATTTGAACGCCAGCACATGGACTCGTCTACTAGCGCAGCTTAATTAACCTAGGCTGCTG
CCACCGCTGAGCAATAACTAGCATAACCCCTTGGGGCCTCTAAACGGGTCTTGAGGGGTTTTTTGCTGAAAGGAGGAACTATATCCGGATTGGCGAATGGGACGCGCCCTGTAGC
GGCGCATTAAGCGCGGCGGGTGTGGTGGTTACGCGCAGCGTGACCGCTACACTTGCCAGCGCCCTAGCGCCCGCTCCTTTCGCTTTCTTCCCTTCCTTTCTCGCCACGTTCGCCG
GCTTTCCCCGTCAAGCTCTAAATCGGGGGCTCCCTTTAGGGTTCCGATTTAGTGCTTTACGGCACCTCGACCCCAAAAAACTTGATTAGGGTGATGGTTCACGTAGTGGGCCATC
GCCCTGATAGACGGTTTTTCGCCCTTTGACGTTGGAGTCCACGTTCTTTAATAGTGGACTCTTGTTCCAAACTGGAACAACACTCAACCCTATCTCGGTCTATTCTTTTGATTTA
TAAGGGATTTTGCCGATTTCGGCCTATTGGTTAAAAAATGAGCTGATTTAACAAAAATTTAACGCGAATTTTAACAAAATATTAACGTTTACAATTTCTGGCGGCACGATGGCAT
GAGATTATCAAAAAGGATCTTCACCTAGATCCTTTTAAATTAAAAATGAAGTTTTAAATCAATCTAAAGTATATATGAGTAAACTTGGTCTGACAGTTACCAATGCTTAATCAGT
GAGGCACCTATCTCAGCGATCTGTCTATTTCGTTCATCCATAGTTGCCTGACTCCCCGTCGTGTAGATAACTACGATACGGGAGGGCTTACCATCTGGCCCCAGTGCTGCAATGA
TACCGCGAGACCCACGCTCACCGGCTCCAGATTTATCAGCAATAAACCAGCCAGCCGGAAGGGCCGAGCGCAGAAGTGGTCCTGCAACTTTATCCGCCTCCATCCAGTCTATTAA
TTGTTGCCGGGAAGCTAGAGTAAGTAGTTCGCCAGTTAATAGTTTGCGCAACGTTGTTGCCATTGCTACAGGCATCGTGGTGTCACGCTCGTCGTTTGGTATGGCTTCATTCAGC
TCCGGTTCCCAACGATCAAGGCGAGTTACATGATCCCCCATGTTGTGCAAAAAAGCGGTTAGCTCCTTCGGTCCTCCGATCGTTGTCAGAAGTAAGTTGGCCGCAGTGTTATCAC
TCATGGTTATGGCAGCACTGCATAATTCTCTTACTGTCATGCCATCCGTAAGATGCTTTTCTGTGACTGGTGAGTACTCAACCAAGTCATTCTGAGAATAGTGTATGCGGCGACC
GAGTTGCTCTTGCCCGGCGTCAATACGGGATAATACCGCGCCACATAGCAGAACTTTAAAAGTGCTCATCATTGGAAAACGTTCTTCGGGGCGAAAACTCTCAAGGATCTTACCG
CTGTTGAGATCCAGTTCGATGTAACCCACTCGTGCACCCAACTGATCTTCAGCATCTTTTACTTTCACCAGCGTTTCTGGGTGAGCAAAAACAGGAAGGCAAAATGCCGCAAAAA
AGGGAATAAGGGCGACACGGAAATGTTGAATACTCATACTCTTCCTTTTTCAATCATGATTGAAGCATTTATCAGGGTTATTGTCTCATGAGCGGATACATATTTGAATGTATTT
AGAAAAATAAACAAATAGGTCATGACCAAAATCCCTTAACGTGAGTTTTCGTTCCACTGAGCGTCAGACCCCGTAGAAAAGATCAAAGGATCTTCTTGAGATCCTTTTTTTCTGC
GCGTAATCTGCTGCTTGCAAACAAAAAAACCACCGCTACCAGCGGTGGTTTGTTTGCCGGATCAAGAGCTACCAACTCTTTTTCCGAAGGTAACTGGCTTCAGCAGAGCGCAGAT
ACCAAATACTGTCCTTCTAGTGTAGCCGTAGTTAGGCCACCACTTCAAGAACTCTGTAGCACCGCCTACATACCTCGCTCTGCTAATCCTGTTACCAGTGGCTGCTGCCAGTGGC
GATAAGTCGTGTCTTACCGGGTTGGACTCAAGACGATAGTTACCGGATAAGGCGCAGCGGTCGGGCTGAACGGGGGGTTCGTGCACACAGCCCAGCTTGGAGCGAACGACCTACA
CCGAACTGAGATACCTACAGCGTGAGCTATGAGAAAGCGCCACGCTTCCCGAAGGGAGAAAGGCGGACAGGTATCCGGTAAGCGGCAGGGTCGGAACAGGAGAGCGCACGAGGGA
GCTTCCAGGGGGAAACGCCTGGTATCTTTATAGTCCTGTCGGGTTTCGCCACCTCTGACTTGAGCGTCGATTTTTGTGATGCTCGTCAGGGGGGCGGAGCCTATGGAAAAACGCC
AGCAACGCGGCCTTTTTACGGTTCCTGGCCTTTTGCTGGCCTTTTGCTCACATGTTCTTTCCTGCGTTATCCCCTGATTCTGTGGATAACCGTATTACCGCCTTTGAGTGAGCTG
ATACCGCTCGCCGCAGCCGAACGACCGAGCGCAGCGAGTCAGTGAGCGAGGAAGCGGAAGAGCGCCTGATGCGGTATTTTCTCCTTACGCATCTGTGCGGTATTTCACACCGCAT
ATATGGTGCACTCTCAGTACAATCTGCTCTGATGCCGCATAGTTAAGCCAGTATACACTCCGCTATCGCTACGTGACTGGGTCATGGCTGCGCCCCGACACCCGCCAACACCCGC
TGACGCGCCCTGACGGGCTTGTCTGCTCCCGGCATCCGCTTACAGACAAGCTGTGACCGTCTCCGGGAGCTGCATGTGTCAGAGGTTTTCACCGTCATCACCGAAACGCGCGAGG
CAGCTGCGGTAAAGCTCATCAGCGTGGTCGTGAAGCGATTCACAGATGTCTGCCTGTTCATCCGCGTCCAGCTCGTTGAGTTTCTCCAGAAGCGTTAATGTCTGGCTTCTGATAA
AGCGGGCCATGTTAAGGGCGGTTTTTTCCTGTTTGGTCACTGATGCCTCCGTGTAAGGGGGATTTCTGTTCATGGGGGTAATGATACCGATGAAACGAGAGAGGATGCTCACGAT
ACGGGTTACTGATGATGAACATGCCCGGTTACTGGAACGTTGTGAGGGTAAACAACTGGCGGTATGGATGCGGCGGGACCAGAGAAAAATCACTCAGGGTCAATGCCAGCGCTTC
GTTAATACAGATGTAGGTGTTCCACAGGGTAGCCAGCAGCATCCTGCGATGCAGATCCGGAACATAATGGTGCAGGGCGCTGACTTCCGCGTTTCCAGACTTTACGAAACACGGA
AACCGAAGACCATTCATGTTGTTGCTCAGGTCGCAGACGTTTTGCAGCAGCAGTCGCTTCACGTTCGCTCGCGTATCGGTGATTCATTCTGCTAACCAGTAAGGCAACCCCGCCA
GCCTAGCCGGGTCCTCAACGACAGGAGCACGATCATGCTAGTCATGCCCCGCGCCCACCGGAAGGAGCTGACTGGGTTGAAGGCTCTCAAGGGCATCGGTCGAGATCCCGGTGCC
TAATGAGTGAGCTAACTTACATTAATTGCGTTGCGCTCACTGCCCGCTTTCCAGTCGGGAAACCTGTCGTGCCAGCTGCATTAATGAATCGGCCAACGCGCGGGGAGAGGCGGTT
TGCGTATTGGGCGCCAGGGTGGTTTTTCTTTTCACCAGTGAGACGGGCAACAGCTGATTGCCCTTCACCGCCTGGCCCTGAGAGAGTTGCAGCAAGCGGTCCACGCTGGTTTGCC
CCAGCAGGCGAAAATCCTGTTTGATGGTGGTTAACGGCGGGATATAACATGAGCTGTCTTCGGTATCGTCGTATCCCACTACCGAGATGTCCGCACCAACGCGCAGCCCGGACTC
GGTAATGGCGCGCATTGCGCCCAGCGCCATCTGATCGTTGGCAACCAGCATCGCAGTGGGAACGATGCCCTCATTCAGCATTTGCATGGTTTGTTGAAAACCGGACATGGCACTC
CAGTCGCCTTCCCGTTCCGCTATCGGCTGAATTTGATTGCGAGTGAGATATTTATGCCAGCCAGCCAGACGCAGACGCGCCGAGACAGAACTTAATGGGCCCGCTAACAGCGCGA
TTTGCTGGTGACCCAATGCGACCAGATGCTCCACGCCCAGTCGCGTACCGTCTTCATGGGAGAAAATAATACTGTTGATGGGTGTCTGGTCAGAGACATCAAGAAATAACGCCGG
AACATTAGTGCAGGCAGCTTCCACAGCAATGGCATCCTGGTCATCCAGCGGATAGTTAATGATCAGCCCACTGACGCGTTGCGCGAGAAGATTGTGCACCGCCGCTTTACAGGCT
TCGACGCCGCTTCGTTCTACCATCGACACCACCACGCTGGCACCCAGTTGATCGGCGCGAGATTTAATCGCCGCGACAATTTGCGACGGCGCGTGCAGGGCCAGACTGGAGGTGG
CAACGCCAATCAGCAACGACTGTTTGCCCGCCAGTTGTTGTGCCACGCGGTTGGGAATGTAATTCAGCTCCGCCATCGCCGCTTCCACTTTTTCCCGCGTTTTCGCAGAAACGTG
GCTGGCCTGGTTCACCACGCGGGAAACGGTCTGATAAGAGACACCGGCATACTCTGCGACATCGTATAACGTTACTGGTTTCACATTCACCACCCTGAATTGACTCTCTTCCGGG
CGCTATCATGCCATACCGCGAAAGGTTTTGCGCCATTCGATGGTGTCCGGGATCTCGACGCTCTCCCTTATGCGACTCCTGCATTAGGAAGCAGCCCAGTAGTAGGTTGAGGCCG
TTGAGCACCGCCGCCGCAAGGAATGGTGCATGCAAGGAGATGGCGCCCAACAGTCCCCCGGCCACGGGGCCTGCCACCATACCCACGCCGAAACAAGCGCTCATGAGCCCGAAGT
GGCGAGCCCGATCTTCCCCATCGGTGATGTCGGCGATATAGGCGCCAGCAACCGCACCTGTGGCGCCGGTGATGCCGGCCACGATGCGTCCGGCGTAGAGGATCGAGATCGATCT
CGATCCCGCGAAATTAATACGACTCACTATA

(D) MHHHHHH**ENLYFQG**NHQIHIHDSDIAFPCAPGQSVLDAALQAGIELPYSCRKGSCGNCASALLDGNITSFNGMAVRSELCTSEQVLLCGCTAASDIRIQPSSFRRLDPEARKRFT
AKVYSNTLAAPDVSLLRLRLPVGKRAKFEAGQYLLIHLDDGESRSYSMANPPHESDGITLHVRHVPGGRFSTIVQQLKSGDTLEIELPFGSIALKPDDTRPLICVAGGTGFAPIK
SVLDDLAKRKVQRDITLIWGARNPSGLYLPSAIDKWRKTWPQFRYIAAITDLGNVPADAHAGRVDDALRTHFGNLHDHVVHCCGSPSLVQSVRTAASDMGLLAQNFHADVFATSP
TGSH*

(E) TGGCGAATGGGACGCGCCCTGTAGCGGCGCATTAAGCGCGGCGGGTGTGGTGGTTACGCGCAGCGTGACCGCTACACTTGCCAGCGCCCTAGCGCCCGCTCCTTTCGCTTTCTTC
CCTTCCTTTCTCGCCACGTTCGCCGGCTTTCCCCGTCAAGCTCTAAATCGGGGGCTCCCTTTAGGGTTCCGATTTAGTGCTTTACGGCACCTCGACCCCAAAAAACTTGATTAGG
GTGATGGTTCACGTAGTGGGCCATCGCCCTGATAGACGGTTTTTCGCCCTTTGACGTTGGAGTCCACGTTCTTTAATAGTGGACTCTTGTTCCAAACTGGAACAACACTCAACCC
TATCTCGGTCTATTCTTTTGATTTATAAGGGATTTTGCCGATTTCGGCCTATTGGTTAAAAAATGAGCTGATTTAACAAAAATTTAACGCGAATTTTAACAAAATATTAACGTTT
ACAATTTCTGGCGGCACGATGGCATGAGATTATCAAAAAGGATCTTCACCTAGATCCTTTTAAATTAAAAATGAAGTTTTAAATCAATCTAAAGTATATATGAGTAAACTTGGTC

TGACAGTTACCAATGCTTAATCAGTGAGGCACCTATCTCAGCGATCTGTCTATTTCGTTCATCCATAGTTGCCTGACTCCCCGTCGTGTAGATAACTACGATACGGGAGGGCTTA
CCATCTGGCCCCAGTGCTGCAATGATACCGCGAGACCCACGCTCACCGGCTCCAGATTTATCAGCAATAAACCAGCCAGCCGGAAGGGCCGAGCGCAGAAGTGGTCCTGCAACTT
TATCCGCCTCCATCCAGTCTATTAATTGTTGCCGGGAAGCTAGAGTAAGTAGTTCGCCAGTTAATAGTTTGCGCAACGTTGTTGCCATTGCTACAGGCATCGTGGTGTCACGCTC
GTCGTTTGGTATGGCTTCATTCAGCTCCGGTTCCCAACGATCAAGGCGAGTTACATGATCCCCCATGTTGTGCAAAAAAGCGGTTAGCTCCTTCGGTCCTCCGATCGTTGTCAGA
AGTAAGTTGGCCGCAGTGTTATCACTCATGGTTATGGCAGCACTGCATAATTCTCTTACTGTCATGCCATCCGTAAGATGCTTTTCTGTGACTGGTGAGTACTCAACCAAGTCAT
TCTGAGAATAGTGTATGCGGCGACCGAGTTGCTCTTGCCCGGCGTCAATACGGGATAATACCGCGCCACATAGCAGAACTTTAAAAGTGCTCATCATTGGAAAACGTTCTTCGGG
GCGAAAACTCTCAAGGATCTTACCGCTGTTGAGATCCAGTTCGATGTAACCCACTCGTGCACCCAACTGATCTTCAGCATCTTTTACTTTCACCAGCGTTTCTGGGTGAGCAAAA
ACAGGAAGGCAAAATGCCGCAAAAAAGGGAATAAGGGCGACACGGAAATGTTGAATACTCATACTCTTCCTTTTTCAATCATGATTGAAGCATTTATCAGGGTTATTGTCTCATG
AGCGGATACATATTTGAATGTATTTAGAAAAATAAACAAATAGGTCATGACCAAAATCCCTTAACGTGAGTTTTCGTTCCACTGAGCGTCAGACCCCGTAGAAAAGATCAAAGGA
TCTTCTTGAGATCCTTTTTTTCTGCGCGTAATCTGCTGCTTGCAAACAAAAAAACCACCGCTACCAGCGGTGGTTTGTTTGCCGGATCAAGAGCTACCAACTCTTTTTCCGAAGG
TAACTGGCTTCAGCAGAGCGCAGATACCAAATACTGTCCTTCTAGTGTAGCCGTAGTTAGGCCACCACTTCAAGAACTCTGTAGCACCGCCTACATACCTCGCTCTGCTAATCCT
GTTACCAGTGGCTGCTGCCAGTGGCGATAAGTCGTGTCTTACCGGGTTGGACTCAAGACGATAGTTACCGGATAAGGCGCAGCGGTCGGGCTGAACGGGGGGTTCGTGCACACAG
CCCAGCTTGGAGCGAACGACCTACACCGAACTGAGATACCTACAGCGTGAGCTATGAGAAAGCGCCACGCTTCCCGAAGGGAGAAAGGCGGACAGGTATCCGGTAAGCGGCAGGG
TCGGAACAGGAGAGCGCACGAGGGAGCTTCCAGGGGGAAACGCCTGGTATCTTTATAGTCCTGTCGGGTTTCGCCACCTCTGACTTGAGCGTCGATTTTTGTGATGCTCGTCAGG
GGGGCGGAGCCTATGGAAAAACGCCAGCAACGCGGCCTTTTTACGGTTCCTGGCCTTTTGCTGGCCTTTTGCTCACATGTTCTTTCCTGCGTTATCCCCTGATTCTGTGGATAAC
CGTATTACCGCCTTTGAGTGAGCTGATACCGCTCGCCGCAGCCGAACGACCGAGCGCAGCGAGTCAGTGAGCGAGGAAGCGGAAGAGCGCCTGATGCGGTATTTTCTCCTTACGC
ATCTGTGCGGTATTTCACACCGCATATATGGTGCACTCTCAGTACAATCTGCTCTGATGCCGCATAGTTAAGCCAGTATACACTCCGCTATCGCTACGTGACTGGGTCATGGCTG
CGCCCCGACACCCGCCAACACCCGCTGACGCGCCCTGACGGGCTTGTCTGCTCCCGGCATCCGCTTACAGACAAGCTGTGACCGTCTCCGGGAGCTGCATGTGTCAGAGGTTTTC
ACCGTCATCACCGAAACGCGCGAGGCAGCTGCGGTAAAGCTCATCAGCGTGGTCGTGAAGCGATTCACAGATGTCTGCCTGTTCATCCGCGTCCAGCTCGTTGAGTTTCTCCAGA
AGCGTTAATGTCTGGCTTCTGATAAAGCGGGCCATGTTAAGGGCGGTTTTTTCCTGTTTGGTCACTGATGCCTCCGTGTAAGGGGGATTTCTGTTCATGGGGGTAATGATACCGA
TGAAACGAGAGAGGATGCTCACGATACGGGTTACTGATGATGAACATGCCCGGTTACTGGAACGTTGTGAGGGTAAACAACTGGCGGTATGGATGCGGCGGGACCAGAGAAAAAT
CACTCAGGGTCAATGCCAGCGCTTCGTTAATACAGATGTAGGTGTTCCACAGGGTAGCCAGCAGCATCCTGCGATGCAGATCCGGAACATAATGGTGCAGGGCGCTGACTTCCGC
GTTTCCAGACTTTACGAAACACGGAAACCGAAGACCATTCATGTTGTTGCTCAGGTCGCAGACGTTTTGCAGCAGCAGTCGCTTCACGTTCGCTCGCGTATCGGTGATTCATTCT
GCTAACCAGTAAGGCAACCCCGCCAGCCTAGCCGGGTCCTCAACGACAGGAGCACGATCATGCTAGTCATGCCCCGCGCCCACCGGAAGGAGCTGACTGGGTTGAAGGCTCTCAA
GGGCATCGGTCGAGATCCCGGTGCCTAATGAGTGAGCTAACTTACATTAATTGCGTTGCGCTCACTGCCCGCTTTCCAGTCGGGAAACCTGTCGTGCCAGCTGCATTAATGAATC
GGCCAACGCGCGGGGAGAGGCGGTTTGCGTATTGGGCGCCAGGGTGGTTTTTCTTTTCACCAGTGAGACGGGCAACAGCTGATTGCCCTTCACCGCCTGGCCCTGAGAGAGTTGC
AGCAAGCGGTCCACGCTGGTTTGCCCCAGCAGGCGAAAATCCTGTTTGATGGTGGTTAACGGCGGGATATAACATGAGCTGTCTTCGGTATCGTCGTATCCCACTACCGAGATGT
CCGCACCAACGCGCAGCCCGGACTCGGTAATGGCGCGCATTGCGCCCAGCGCCATCTGATCGTTGGCAACCAGCATCGCAGTGGGAACGATGCCCTCATTCAGCATTTGCATGGT
TTGTTGAAAACCGGACATGGCACTCCAGTCGCCTTCCCGTTCCGCTATCGGCTGAATTTGATTGCGAGTGAGATATTTATGCCAGCCAGCCAGACGCAGACGCGCCGAGACAGAA
CTTAATGGGCCCGCTAACAGCGCGATTTGCTGGTGACCCAATGCGACCAGATGCTCCACGCCCAGTCGCGTACCGTCTTCATGGGAGAAAATAATACTGTTGATGGGTGTCTGGT
CAGAGACATCAAGAAATAACGCCGGAACATTAGTGCAGGCAGCTTCCACAGCAATGGCATCCTGGTCATCCAGCGGATAGTTAATGATCAGCCCACTGACGCGTTGCGCGAGAAG
ATTGTGCACCGCCGCTTTACAGGCTTCGACGCCGCTTCGTTCTACCATCGACACCACCACGCTGGCACCCAGTTGATCGGCGCGAGATTTAATCGCCGCGACAATTTGCGACGGC
GCGTGCAGGGCCAGACTGGAGGTGGCAACGCCAATCAGCAACGACTGTTTGCCCGCCAGTTGTTGTGCCACGCGGTTGGGAATGTAATTCAGCTCCGCCATCGCCGCTTCCACTT
TTTCCCGCGTTTTCGCAGAAACGTGGCTGGCCTGGTTCACCACGCGGGAAACGGTCTGATAAGAGACACCGGCATACTCTGCGACATCGTATAACGTTACTGGTTTCACATTCAC
CACCCTGAATTGACTCTCTTCCGGGCGCTATCATGCCATACCGCGAAAGGTTTTGCGCCATTCGATGGTGTCCGGGATCTCGACGCTCTCCCTTATGCGACTCCTGCATTAGGAA
GCAGCCCAGTAGTAGGTTGAGGCCGTTGAGCACCGCCGCCGCAAGGAATGGTGCATGCAAGGAGATGGCGCCCAACAGTCCCCCGGCCACGGGGCCTGCCACCATACCCACGCCG
AAACAAGCGCTCATGAGCCCGAAGTGGCGAGCCCGATCTTCCCCATCGGTGATGTCGGCGATATAGGCGCCAGCAACCGCACCTGTGGCGCCGGTGATGCCGGCCACGATGCGTC
CGGCGTAGAGGATCGAGATCGATCTCGATCCCGCGAAATTAATACGACTCACTATAGGGGAATTGTGAGCGGATAACAATTCCCCTCTAGAAATAATTTTGTTTAACTTTAAGAA
GGAGATATACCATGGcATGCATCACCACCACCACCACGAGAACCTGTACTTCCAGGGTaaccaccaaatccacattcacgatagcgacatcgcgtttccgtgcgcgccgggtca
gagcgtgctggacgcggcgctgcaagcgggcattgaactgccgtatagctgccgtaaaggtagctgcggtaactgcgcgagcgcgctgctggatggtaacatcaccagcttcaac
ggcatggcggtgcgtagcgagctgtgcaccagcgaacaagttctgctgtgcggttgcaccgcggcgagcgacatccgtattcaaccgagcagcttccgtcgtctggacccggagg
cgcgtaagcgttttaccgcgaaagtttacagcaacaccctggcggcgccggatgtgagcctgctgcgtctgcgtctgccggttggcaagcgtgcgaaatttgaagcgggccagta
cctgctgattcacctggacgatggcgagagccgtagctatagcatggcgaacccgccgcacgaaagcgacggcatcaccctgcatgtgcgtcatgttccgggtggccgtttcagc
accattgtgcagcaactgaagagcggtgataccctggagatcgaactgccgtttggcagcattgcgctgaaaccggacgatacccgtccgctgatctgcgttgcgggtggcaccg
gttttgcgccgattaaaagcgtgctggacgatctggcgaagcgtaaagttcagcgtgacatcaccctgatttggggtgcgcgtaacccgagcggtctgtacctgccgagcgcgat
cgataagtggcgtaaaacctggccgcaattccgttatatcgcggcgattaccgacctgggtaacgtgccggcggatgcgcatgcgggtcgtgttgacgatgcgctgcgtacccac
tttggtaacctgcacgatcacgtggttcattgctgcggcagcccgagcctggtgcagagcgttcgtaccgcggcgagcgacatgggtctgctggcgcaaaacttccacgcggatg
tgtttgcgaccagcccgaccggcagccactaaCTCGAGTCTGGTAAAGAAACCGCTGCTGCGAAATTTGAACGCCAGCACATGGACTCGTCTACTAGCGCAGCTTAATTAACCTA
GGCTGCTGCCACCGCTGAGCAATAACTAGCATAACCCCTTGGGGCCTCTAAACGGGTCTTGAGGGGTTTTTTGCTGAAAGGAGGAACTATATCCGGAT

Figure S3. Sequences used in this work. Amino acid sequences of the **α (A)** and **β (B)** subunits of the wild-type dioxygenase (*Comamonas* sp. E6), with TEV protease recognition site in bold, the hexahistidine tag underlined, and stop codon represented with a *. **(C)** Nucleotide sequence for the TPADO construct, with codons for the TEV protease recognition site and 6xHis tag in bold, and the codons for the insert genes underlined and in lower case. **(D)** Amino acid and **(E)** nucleotide sequences for the reductase and its vector construct, respectively.

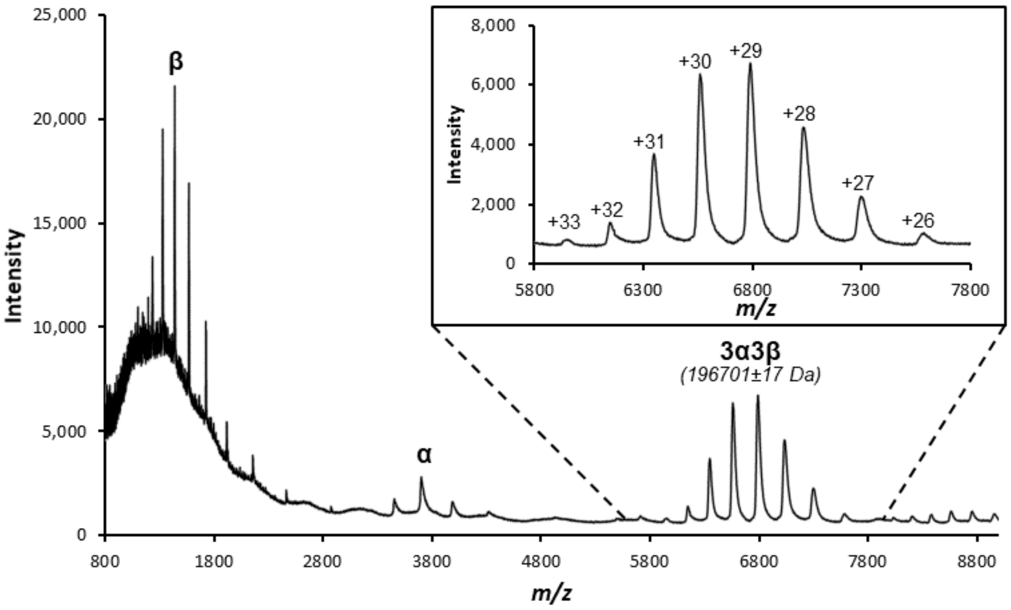

Figure S4. Native mass spectrum of TPADO. The protein has a charge envelope from +26 to +33 with an expected Gaussian distribution. The native species (spectrum shown on an amplified scale in the inset) had a deconvoluted mass of 196,701 Da corresponding to the native 3α3β complex. This is within 0.5% error of the theoretical mass of 196,012, as predicted by the online ExPasy MW calculator tool (10).

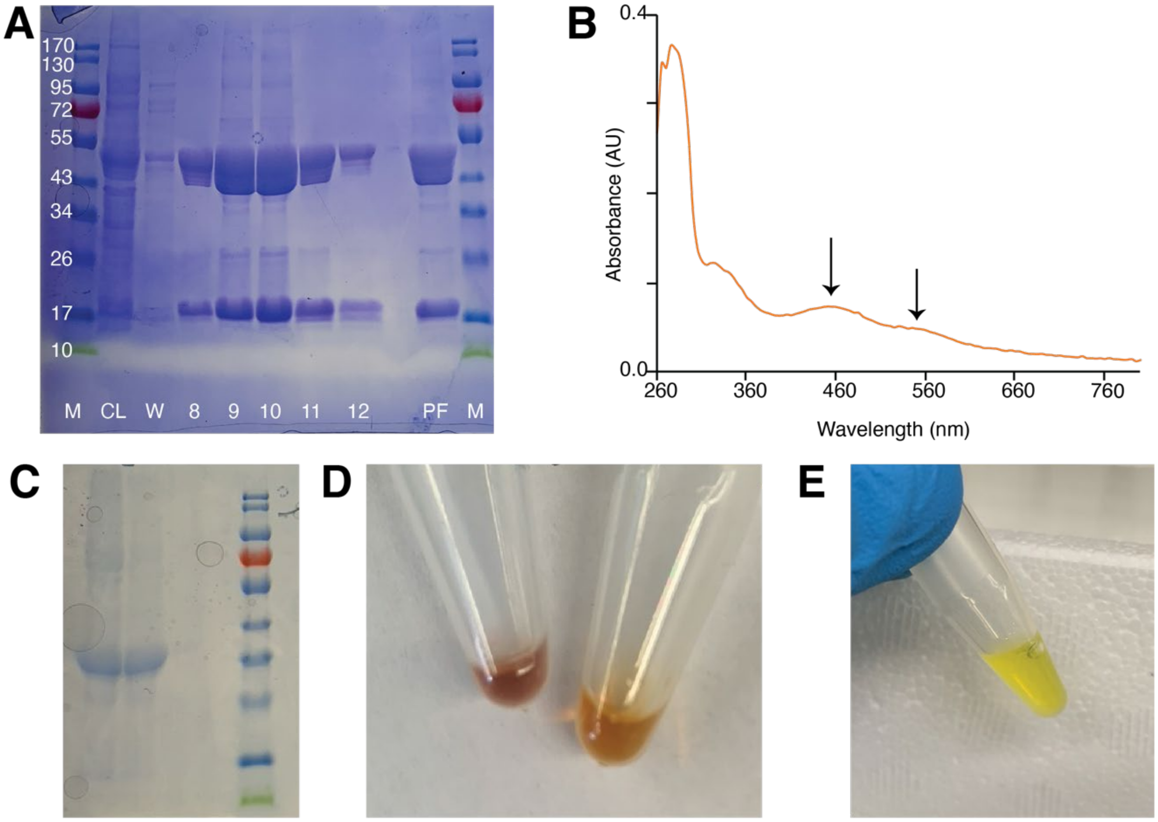

Figure S5. Isolated TPADO and reductase reflect high yield purification and the presence of cofactors. (A) SDS-PAGE and (B) UV/visible spectra of TPADO. SDS-PAGE of a molecular weight size marker (M, in kDa), clarified lysate (CL), wash (W), and Ni-NTA column eluate fraction (8-12) clearly indicates enrichment in the heavier alpha and lighter beta subunits of TPADO. The pooled fractions (PF) obtained were buffer exchanged to remove imidazole (>95% purity) and were used for the production of crystals. UV/visible solution spectrum of pure TPADO shows the expected peak maxima at 450 and 550 nm for a [2Fe-2S] cluster and mononuclear non-heme iron, respectively. The peak at ~300 nm may also be attributed to the mononuclear non-heme iron. Atomic absorption carried out on 3 independent preparations of the TPADO enzyme contained 3.5 (± 0.08), 3.1 (± 0.05), and 2.9 (± 0.06) atoms of iron per TPADO $\alpha\beta$ monomer, where the latter protein concentration was determined by Bradford assay for the complex of known molecular weight. Errors represent one standard deviation based on averaged measurements of 3 individual dilutions. (C) SDS-PAGE of reductase, showing a band near the expected mass of 36 kDa. (D) Samples of TPADO (left) and reductase (right). (E) Reductase protein was precipitated with 3% trichloroacetic acid (w/v %), and yellow flavin was extracted from the soluble portion.

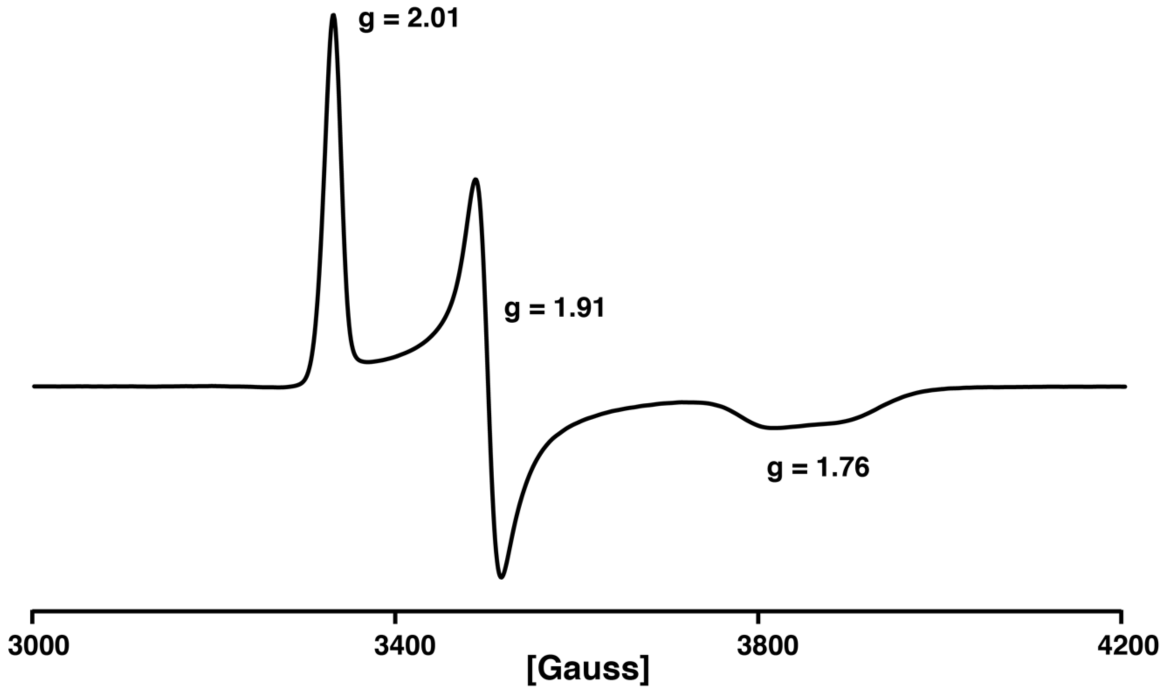

Figure S6. EPR signal from the dithionite treated (reduced) Rieske cluster of TPADO that displays the characteristic features (g = 2.01, 1.91, and 1.76) of this cofactor. Conditions: microwave power, 2.0 mW; temperature, 50 K; microwave frequency, 100 KHz (11).

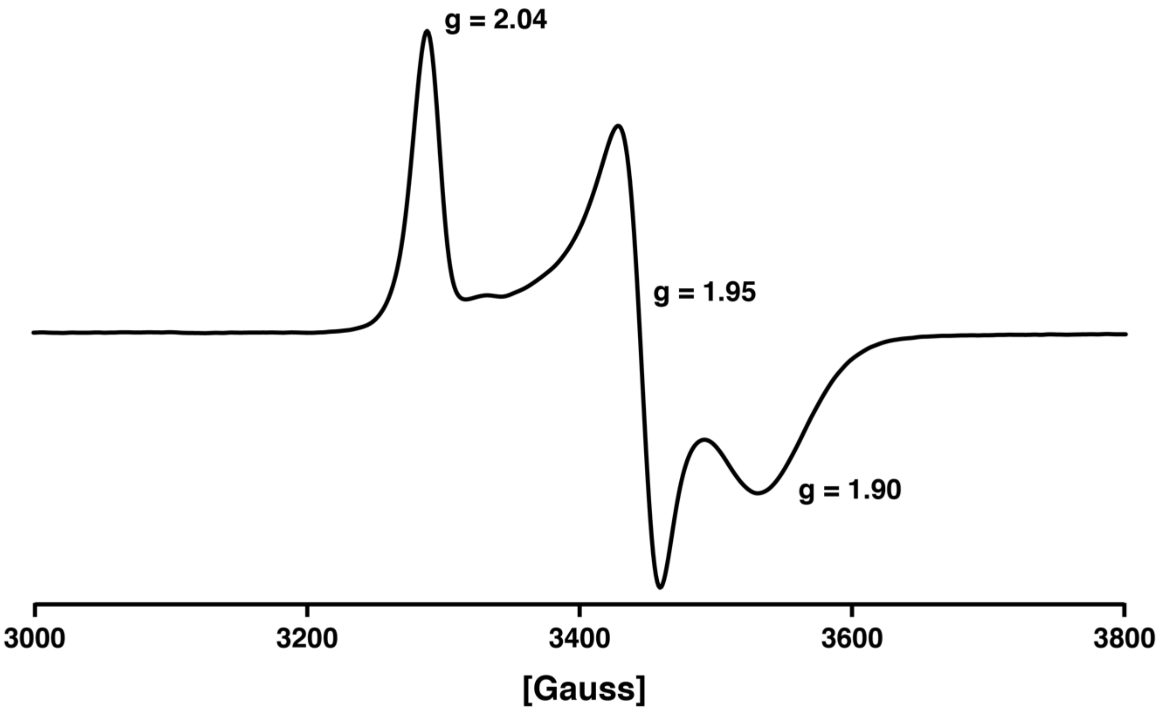

Figure S7. EPR signal from the NADH treated (reduced) Plant-type cluster of the reductase, displaying the characteristic features of this cofactor (g = 2.04, 1.95, and 1.90). Conditions: microwave power, 2.0 mW; temperature, 50 K; microwave frequency, 100 KHz (12).

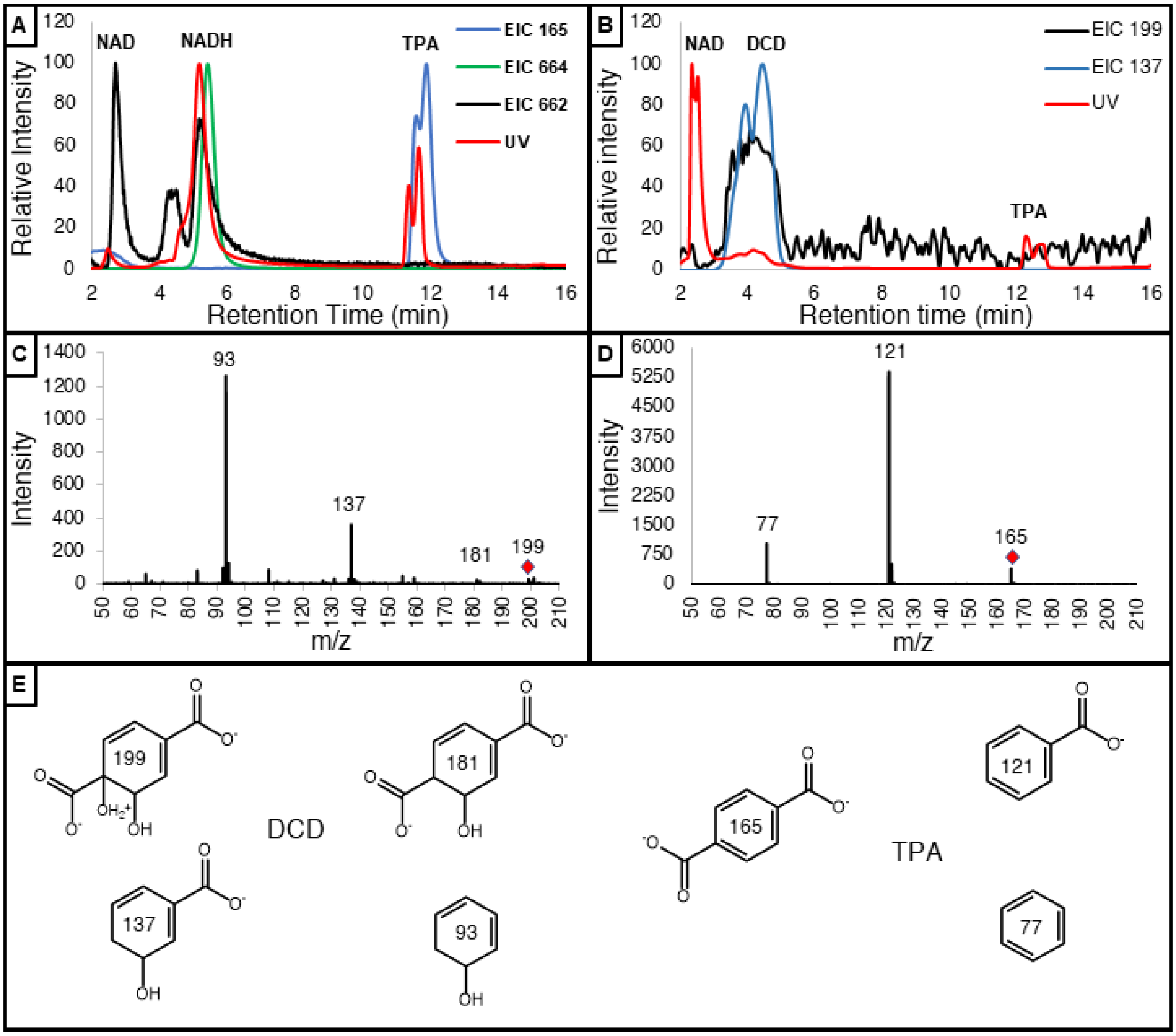

Figure S8. Extracted ion chromatograms and tandem mass spectra of TPA and DCD ions. (A) Extracted ion chromatograms (EIC) of a reaction mixture containing TPA and NADH, prior to the addition of enzymes. The expected molecular ions for NAD^+ (M-H- m/z = 662), NADH (M-H- m/z = 664) and TPA (M-H- m/z = 165) were detected. Further investigation of the spectra containing the TPA molecular ion revealed a coeluting ion of greater intensity with an m/z = 121 corresponding to loss of CO_2 (Δm=44). There is no observable peak corresponding to the molecular ion of DCD. Overlaying the UV/vis chromatogram indicates the detectability of TPA, NADH, and NAD^+. **(B)** EICs for a reaction mixture containing TPA and NADH following incubation with TPADO and reductase for 90 minutes. The same three ions, plus a species at m/z 199 which is the expected mass of the molecular ion of DCD (M-H- m/z = 199), were detected. The intensity of the ion at m/z 199 was low; however, a series of ions was observed to co-elute, the most intense ion being m/z = 137. This ion was hypothesized to come from in-source fragmentation leading to the loss of H_2O + CO_2 (Δm=62) from DCD. **(C)** A tandem mass spectrum of the parent ion of DCD (diamond), showing a fragmentation pattern consistent with the fragments observed in source ionization in the EIC above. **(D)** A tandem mass spectrum of the parent ion of TPA (diamond), showing a fragmentation pattern consistent with the fragments observed in source ionization in the EIC above. **(E)** Predicted fragment ions for DCD (left) and TPA (right). m/z values are inscribed inside each molecule. See Table S1 for exact masses of all ion species described here.

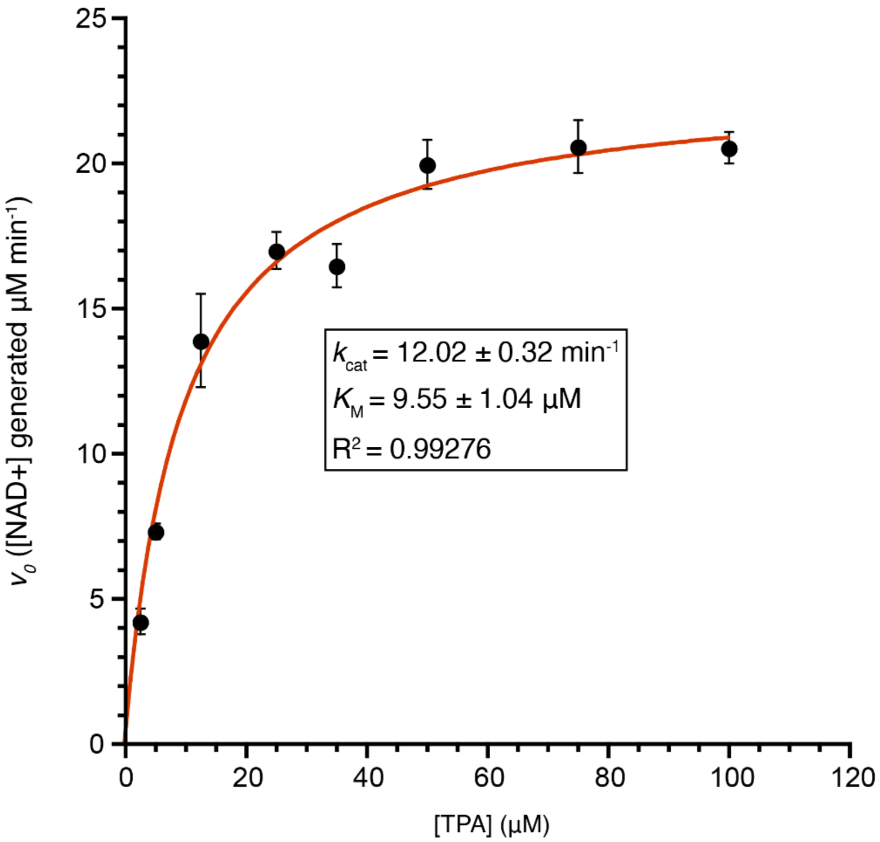

Figure S9. Apparent steady-state kinetic parameters of TPADO. Buffered solutions of TPADO (1.9 µM), reductase (0.6 µM), and NADH (200 µM) were reacted with varying concentrations of terephthalate (2.5–100 µM) in a quartz cuvette (1 cm path length, concentrations after mixing). Solutions were prepared in ambient air (~230 µM O_2) and contained 20 mM Tris, 150 mM NaCl, 20% DMSO (v/v), 10% glycerol (v/v), pH 8.0 buffer at 28 ºC. The initial rates of reactions (v_0) were obtained from the slope of the linear, first 5-10% of the time course using the extinction coefficient for NADH at 340 nm (6.22 mM^{-1} x cm^{-1}). Averages of 3 rates are plotted with error bars representing ±1 standard deviation. The red line is a fit of the data to the Michaelis-Menten hyperbolic expression.

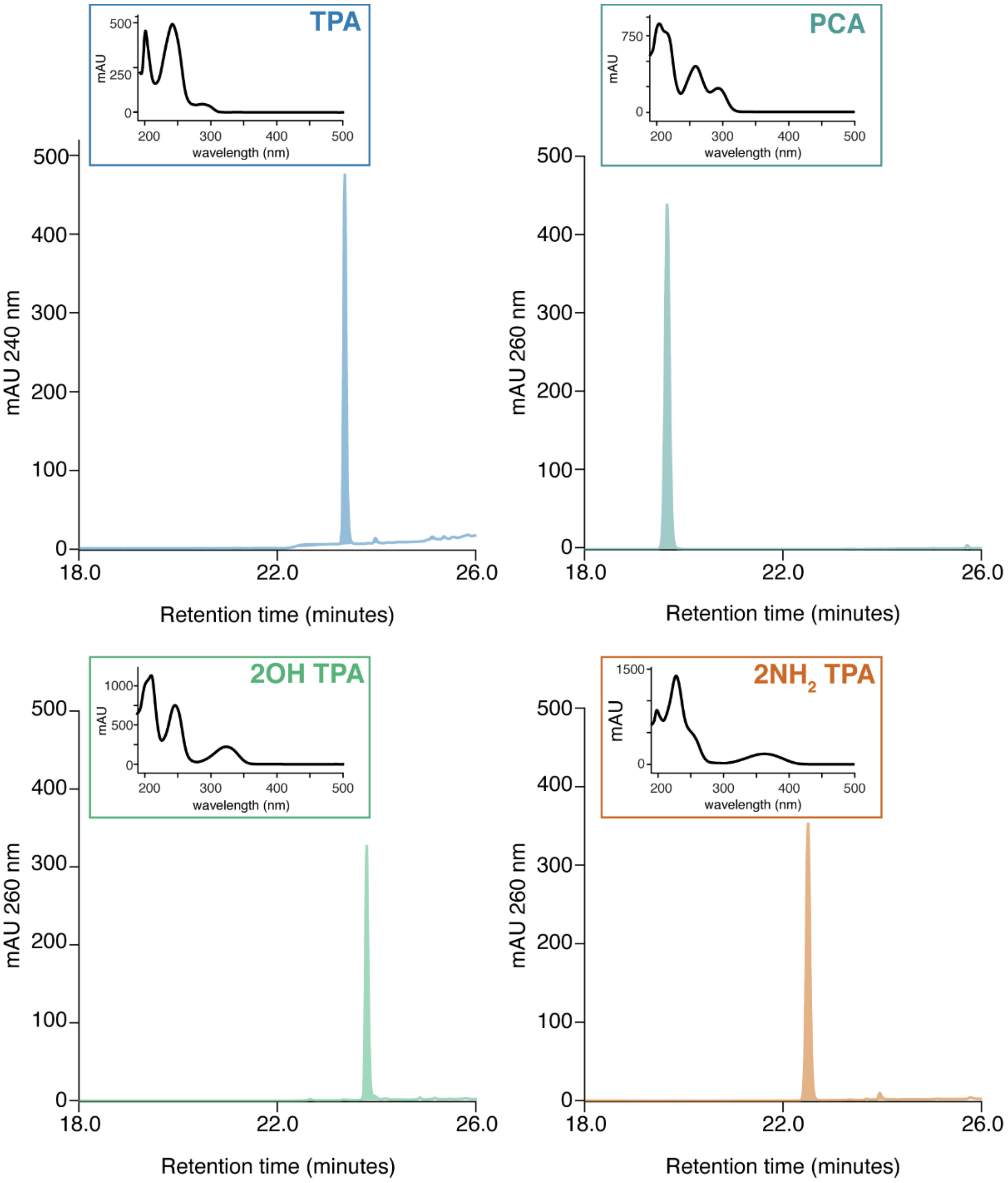

Figure S10. Standards samples of (A) TPA, (B) PCA, (C) 2-OH-TPA, and (D) 2NH$_2$-TPA resolved via reverse-phase HPLC according to the program described in *Detailed experimental methods*. Retention times are given in Table S3.

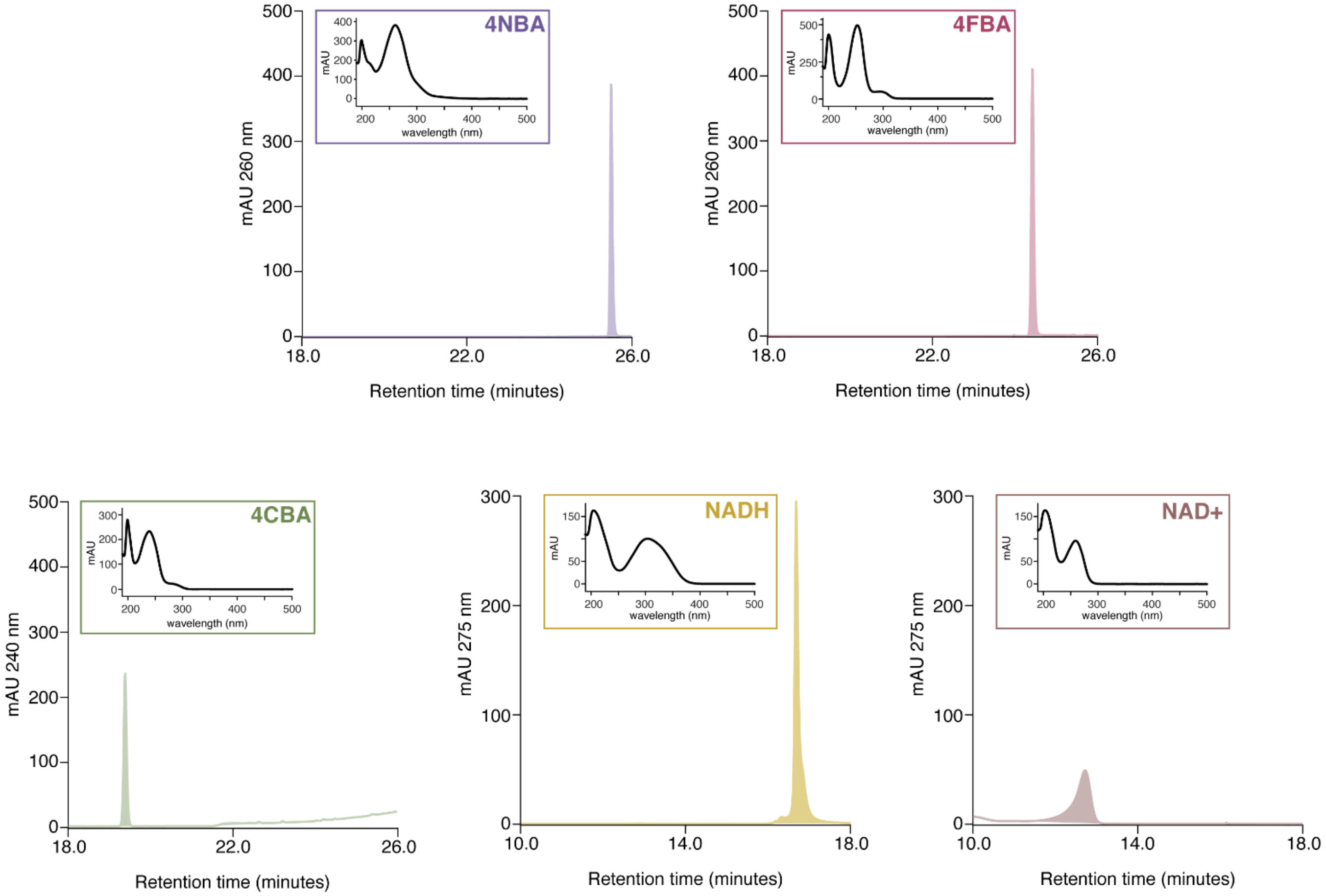

Figure S11. Standards of (A) 4-NBA, (B) 4-FBA, (C) 4-CBA, (D) NADH, and (E) NAD+ resolved via reverse-phase HPLC according to the program described in *Detailed experimental methods*. Retention times are given in Table S3.

\

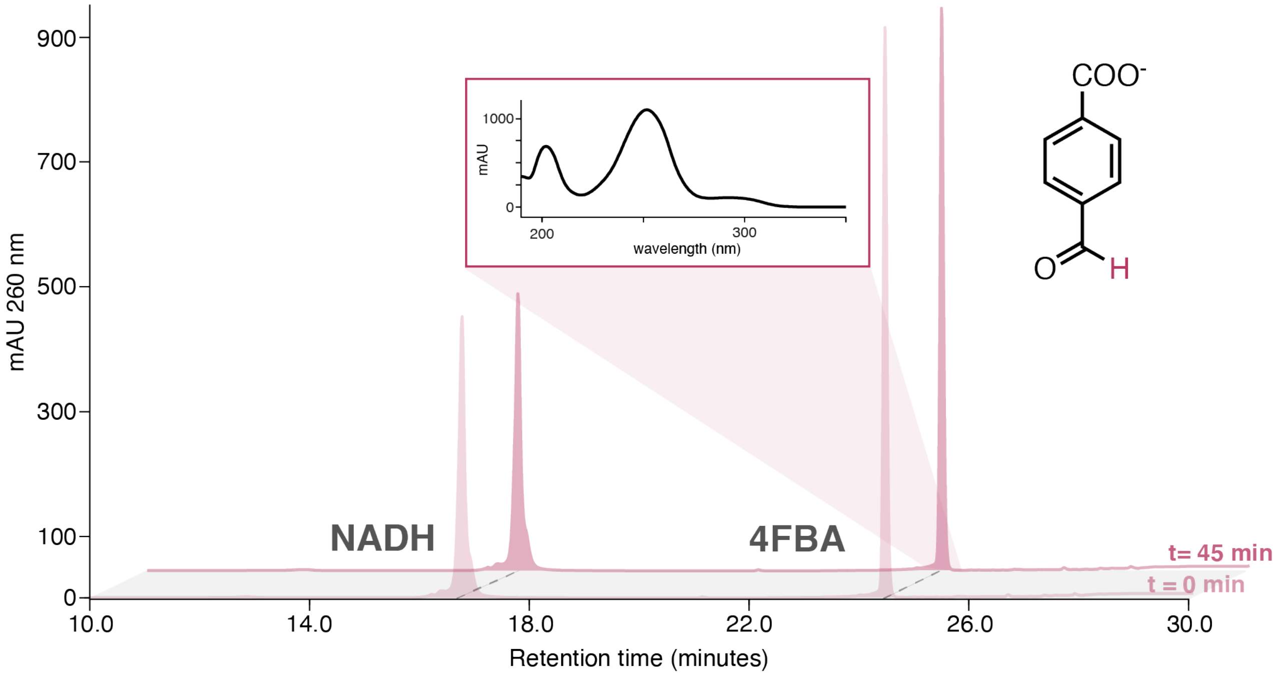

Figure S12. HPLC chromatograms monitoring 4-formylbenzoate (4FBA) and NADH before and after incubation with TPADO/reductase. Front, t = 0 sample. Back, sample quenched at 45 minutes. Inset, extracted UV/vis spectrum from the 4FBA peak. There were no changes in the NADH and 4FBA peak areas that were above background. Additionally, no quantifiable NAD+ production was observed.

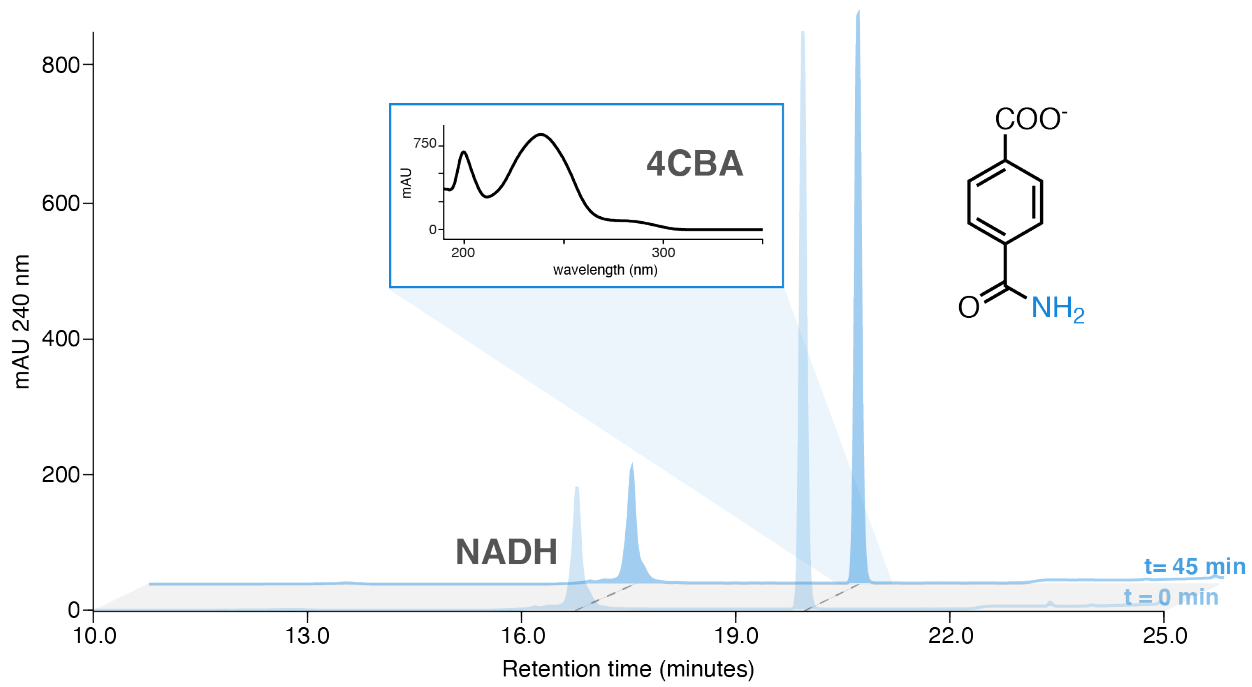

Figure S13. HPLC chromatograms monitoring the 4-carbamoylbenzoate (4CBA) and NADH before and after incubation with TPADO/reductase. Front, t = 0 sample. Back, sample quenched at 45 minutes. Inset, extracted UV/vis spectrum from the 4CBA peak. There were no changes in the NADH and 4CBA peak areas that were above background. Additionally, no quantifiable NAD+ production was observed.

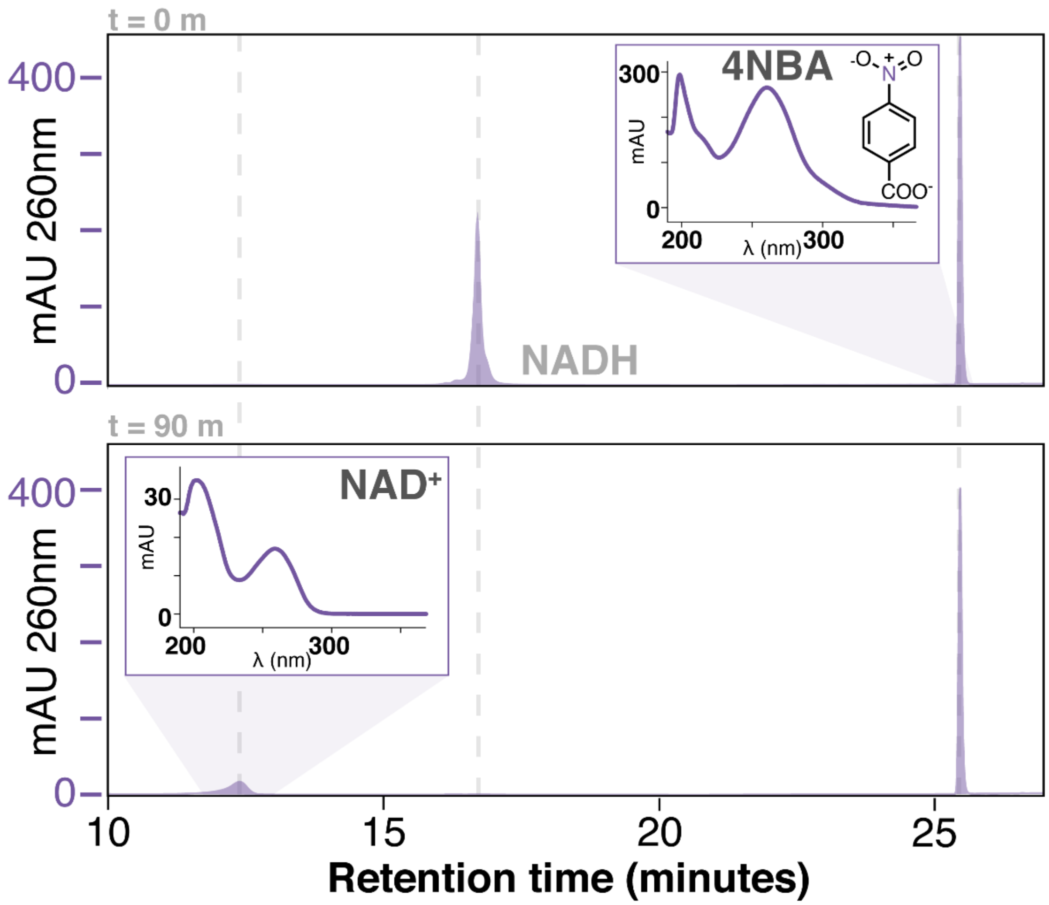

Figure S14. HPLC chromatograms monitoring 4-nitrobenzoate (4-NBA) and NADH before (top) and after incubation with TPADO/reductase. Front, t = 0 sample. Back, sample quenched at 90 minutes. These data indicate the complete conversion of NADH to NAD^+ in the presence of enzyme and 4-NBA, without the formation of any 4-NBA associated product.

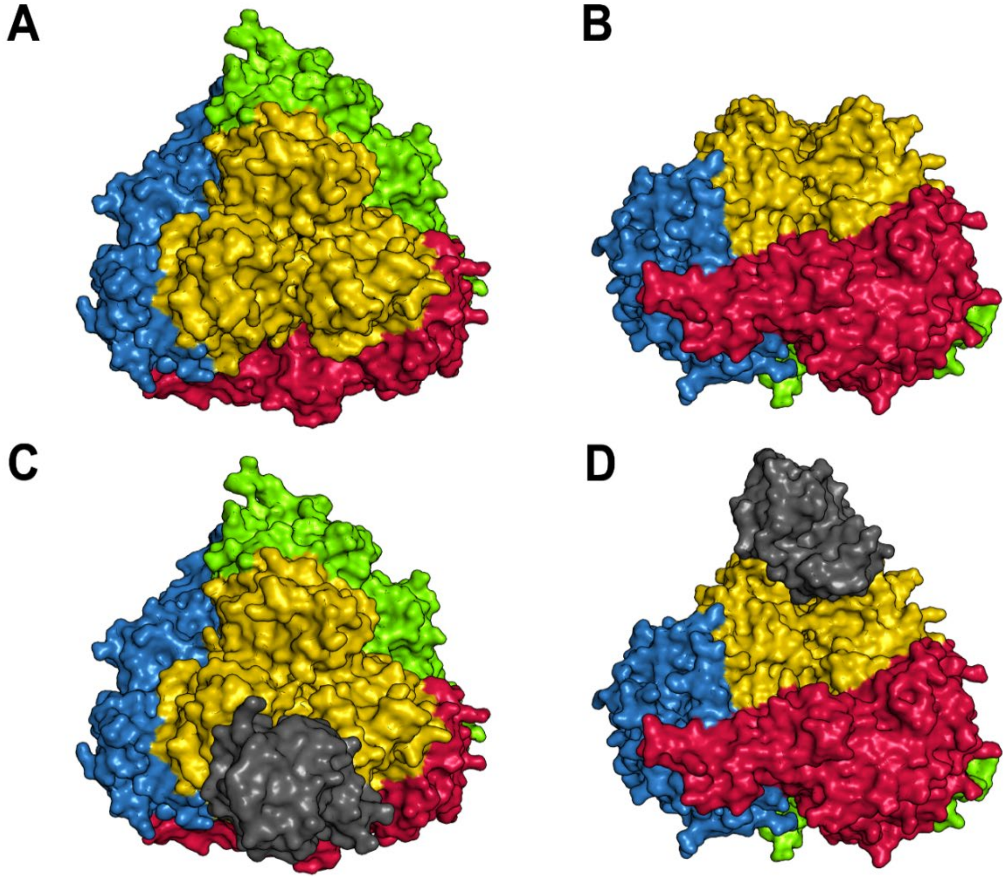

Figure S15. Lysozyme binding to the TPADO structure. A) Top and B) Side view of the TPADO $\alpha_3\beta_3$-heterohexamer. The α-subunits are colored in red, green and blue. The β-subunits are colored in yellow. C) Top and D) Side view of the TPADO $\alpha_3\beta_3$-heterohexamer including the bound lysozyme protein in gray color.

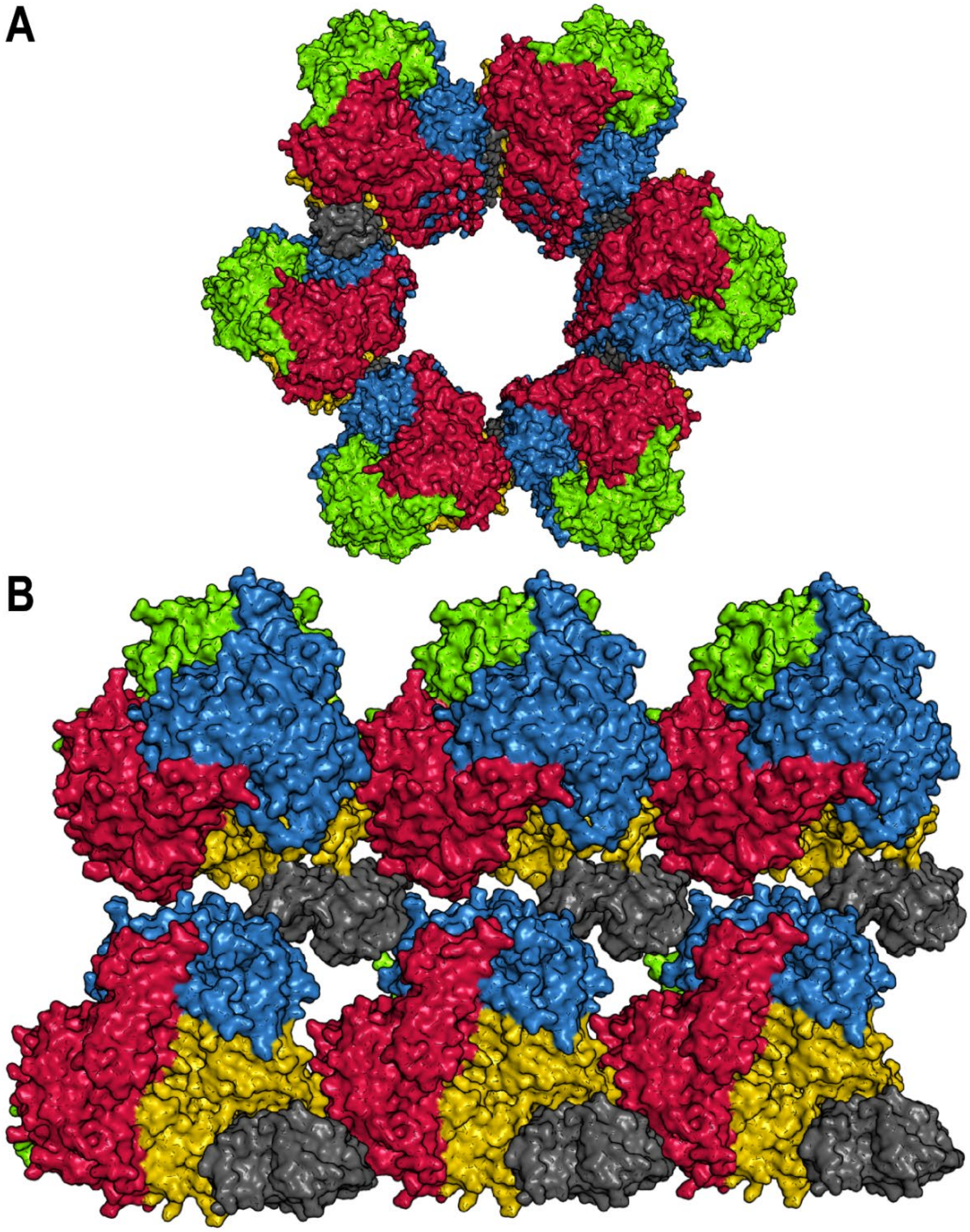

Figure S16. TPADO crystal packing interactions viewed A) along the hexagonal hole and B) inside the hexagonal hole show the influence of lysozyme (gray) as a crystallization chaperone to form stable crystal contacts.

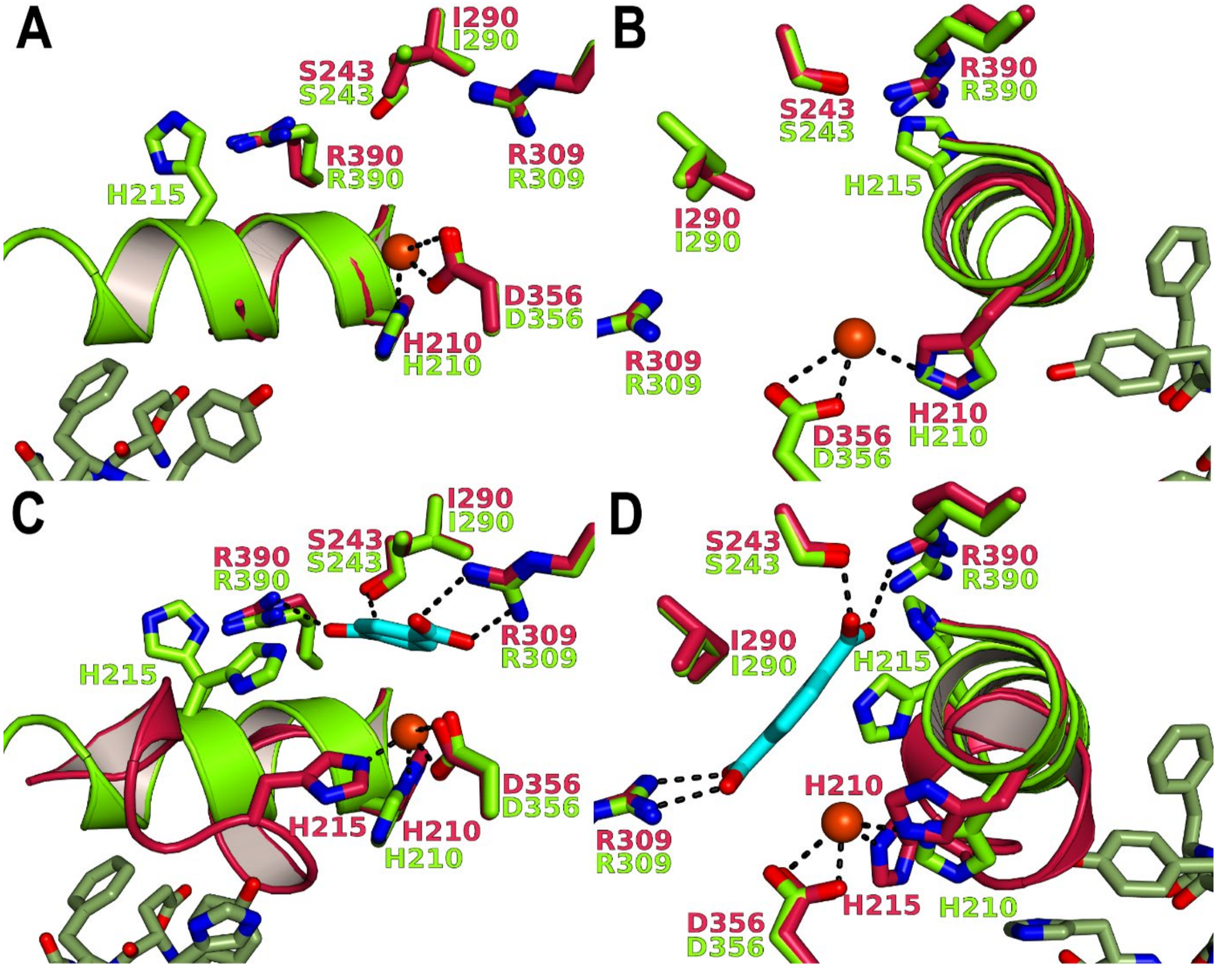

Figure S17. Superposition of the catalytic active sites of two TPADO α-subunits within the ligand-free state (A and B) as well as for the crystal structures of the TPA bound state (C and D). For the light green colored α-subunit, an α-helix with residues 208 to 220 is stabilized by crystal packing interactions (residues of a neighboring symmetry related molecule are shown in dark green). The ferrous ion coordinating histidine residues H210 and H215 are located on this α-helix. In the ligand-free state, the non-stabilized α-helix within the red colored α-subunit adopts the same α-helical conformation as the stabilized green α-helix, but is disordered from residue H215 onwards. Consequently, residue H215 does not coordinate the ferrous ion in the green colored α-subunit. The H210 side chain residues of both the red and green colored α-subunits align very well. In the TPA-bound state, the non-stabilized red α-helix unfolds upon TPA binding and enables the coordination of the H215 side chain with the ferrous ion. For residue H210, the peptide backbone still aligns very well between both α-subunits, but the side chain conformation differs to avoid steric clashes with residues of the unfolded helix. Substrate TPA is shown in light blue. Hydrogen bonding interactions are only shown for the red colored α-subunit for both the Apo and the TPA-bound state.

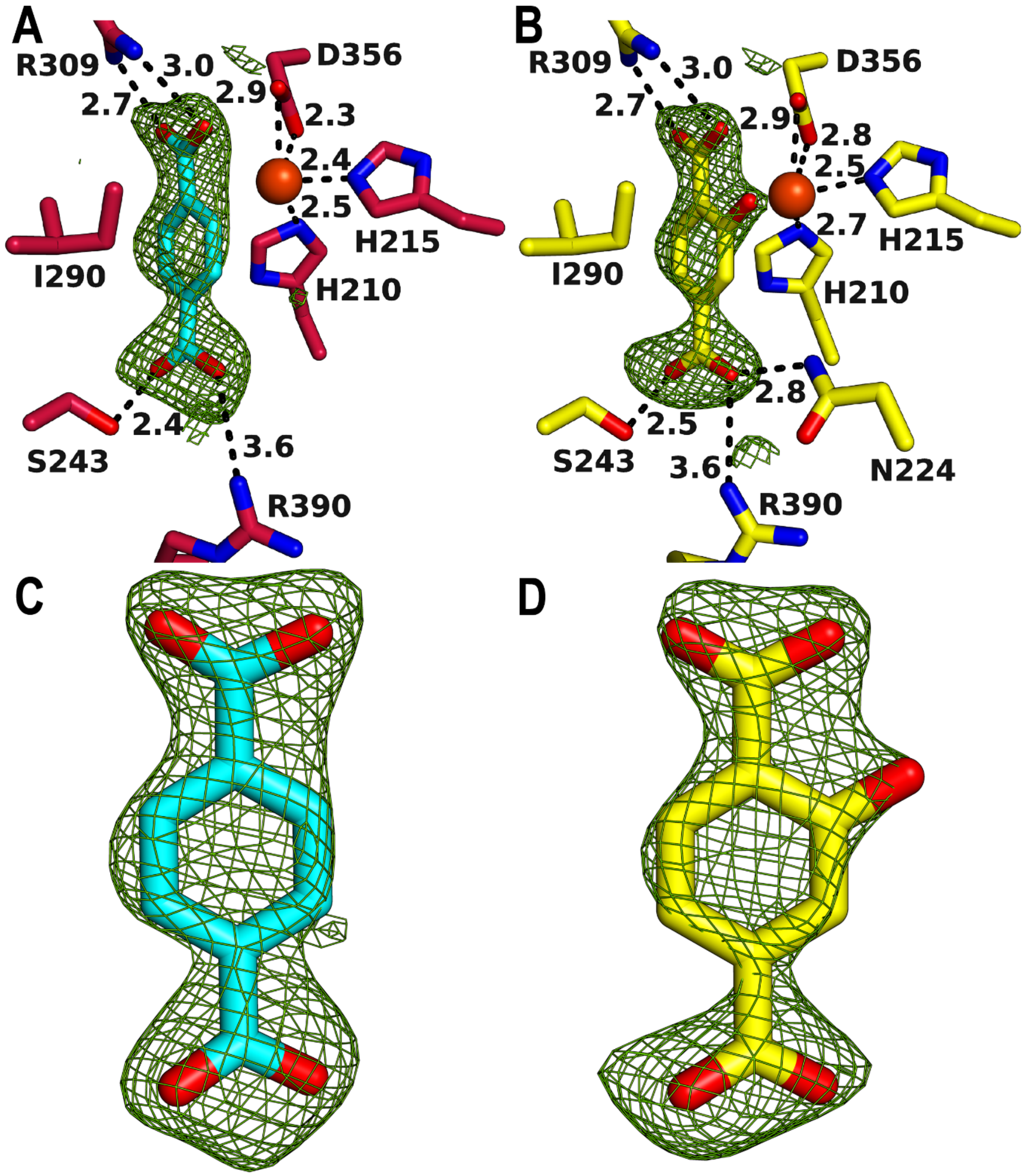

Figure S18. Omit electron density maps for the TPADO bound substrates. Active site interactions of the A) TPA and B) 2-OH_TPA bound structure with omit electron density maps shown for the substrates at 2.5 σ level. Zoom on the omit electron density maps of substrates C) TPA and D) 2-OH_TPA.

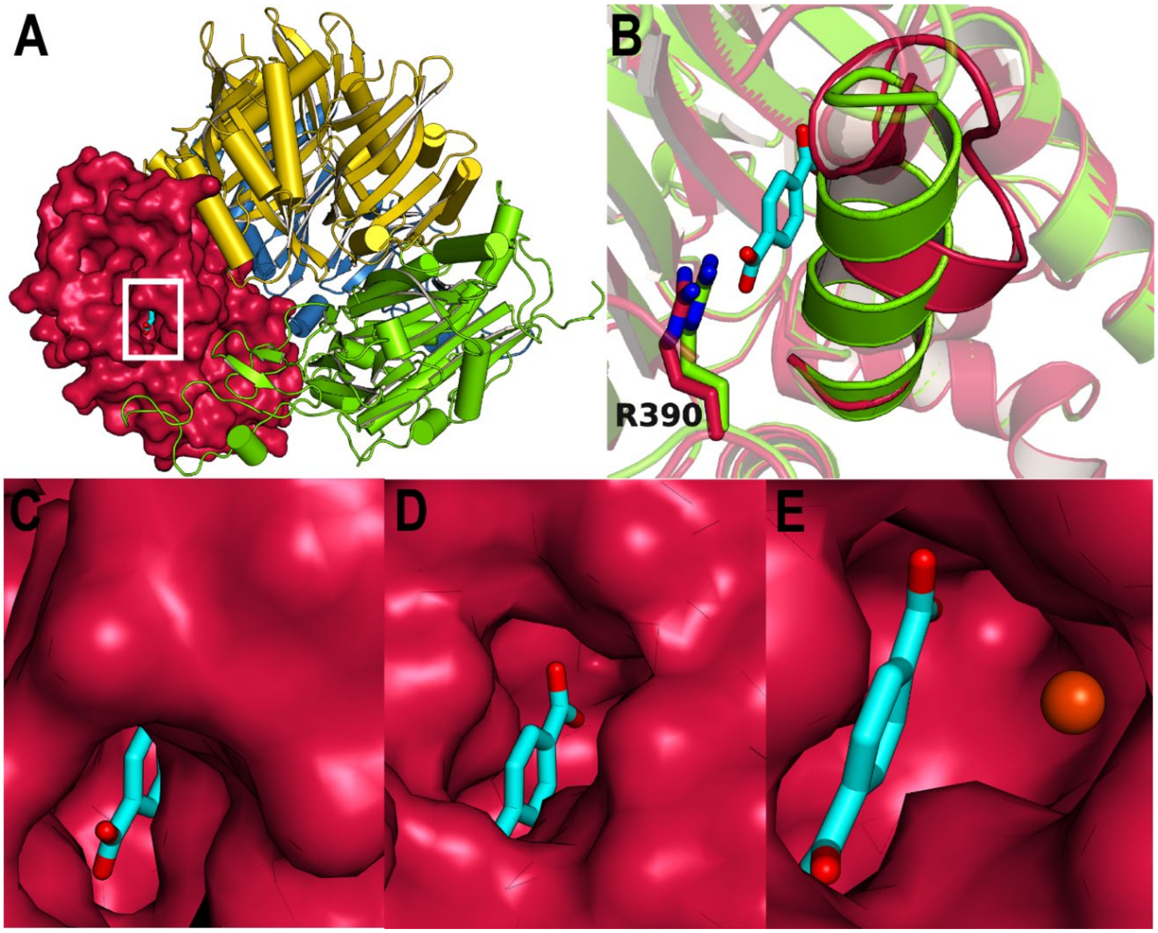

Figure S19. Visualization of a potential substrate entry / product release site of TPADO. A) Side view of the TPADO $\alpha_3\beta_3$-heterohexamer shows the red colored α-subunit as surface presentation to locate a potential substrate entry site for TPA (white square). TPA is colored in light blue. B) Superposition of the green and red colored α-subunits show that substrate access is gated by arginine residue R390 as well as the conformation of an α-helix which contains the ferrous ion coordinating histidine residues H210 and H215. C) A solvent accessible surface rendering of the TPA-bound structure reveals that TPA is accommodated in a deep and partially closed pocket (area shown represents the white square from A). The active site of the TPA-bound state is partially accessible due to the flexible residues 223 to 226 which were not visible within the electron density. D and E) Two alternative viewing angles are shown of the Apo structure with TPA and the ferrous ion modelled from the TPA bound state. The TPA binding pocket is more open within the substrate-free structure because the α-helical residues from H215 onwards are disordered and not part of the model (in total residues 215 to 227 are missing). The TPA binding pocket is completely closed within the green colored α-subunit as well as for the red-colored α-subunit of the 2-OH-TPA-bound structure in which all residues are defined within the electron density (not shown)

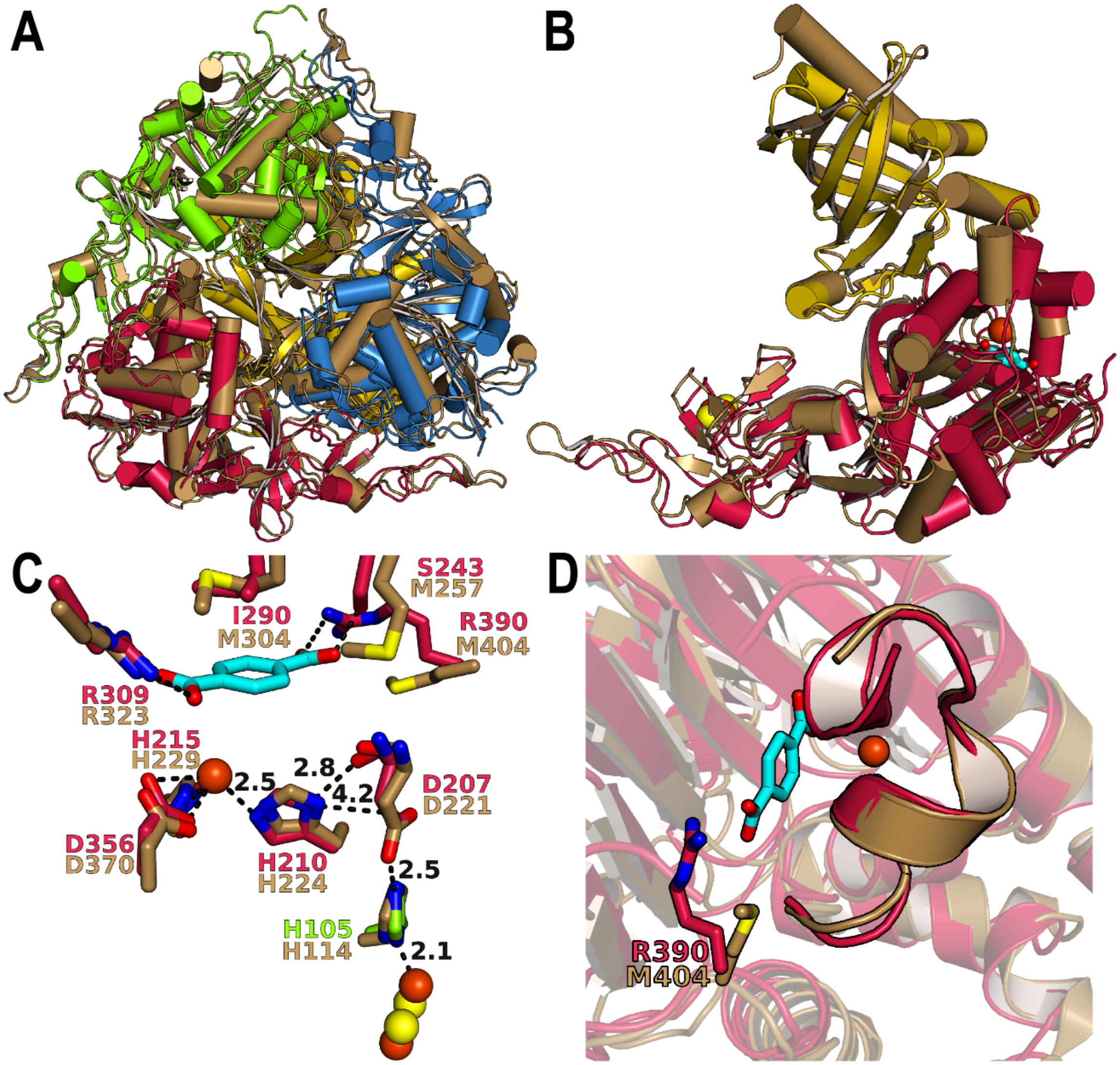

Figure S20. Structural comparison of TPADO with its closest structural homolog salicylate 5-monooxygenase (NagGH). The TPA-bound red colored TPADO α-subunit was superimposed to a NagGH α-subunit (pdb 7C8Z). A) Bottom view of the superimposed $\alpha_3\beta_3$-heterohexamers shows that the overall quaternary architecture between TPADO and NagGH is very similar. The TPADO α-subunits are colored in red, green and blue. The TPADO β-subunits are colored in yellow. NagGH is colored in beige. B) Side view of the superposition shown for one αβ-subunit. C) Zoom into the catalytic active site shows that the residues coordinating the ferrous ion as well as connecting the [2Fe-2S] cluster of a neigboring α-subunit with the ferrous ion align very well. Hydrogen bonds are only shown for TPADO for better visibility. Residue H105 belongs to a neighboring α-subunit and is colored in green. For residue D207, the hydrogen bonding distances of both the carbonyl oxygen and the side chain Oδ-atom are plotted to the Nδ atom of the H210 side chain. Atom distances are shown for the hydrogen bonds connecting the ferrous ion with the [2Fe-2S] cluster. D) The α-helices which contain the ferrous ion coordinating histidine residues align very well between both structures. The substrate entry site has a methionine in NagG in contrast to TPADO which harbors an arginine residue.

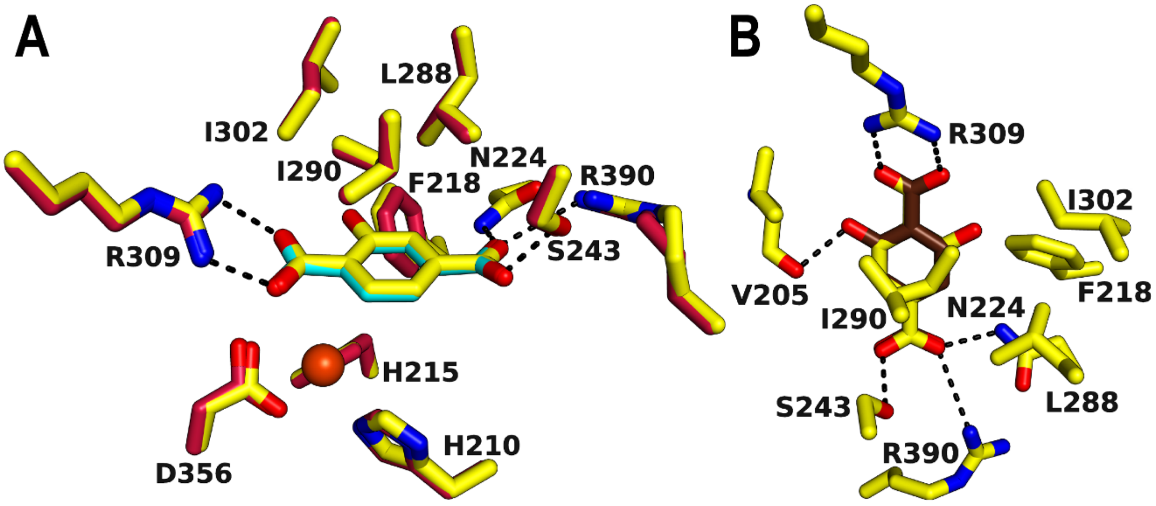

Figure S21: Comparison between the TPA and 2-OH-TPA bound structures. A) Superposition of the 2-OH-TPA-bound TPADO structure (yellow) with the TPA-bound structure (protein in red, TPA in light blue) shows that the ligands and the ligand interacting residues align very well. The 2-hydroxyl group of 2-OH-TPA is oriented towards the hydrophobic part of the catalytic site formed by residues F218, L288, I290 and I302. Residue N224 which is disordered in the TPA structure is ordered in one α-subunit within the 2-OH-TPA bound structure and forms a hydrogen bond with one of 2-OH-TPA's carboxylate groups. B) Ligand 2-OH-TPA (yellow) was flipped (ligand model brown) to show that the 2-hydroxyl group would be able to form a hydrogen bond with the carbonyl oxygen of residue V205 but the electron density strongly suggests that the hydroxyl-group is oriented towards the hydrophobic side instead.

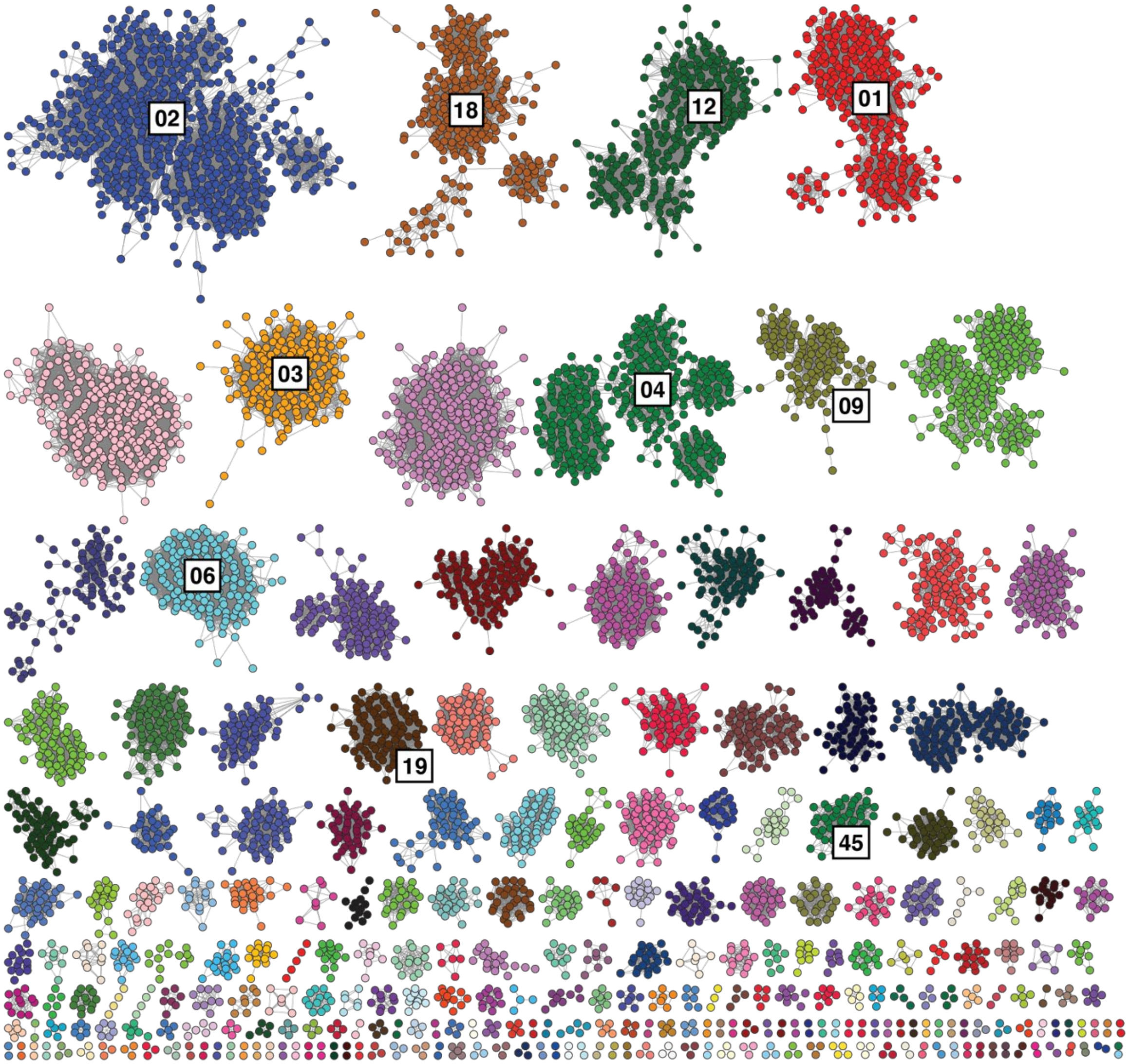

Figure S22. Color sequence similarity network of alpha subunit proteins from aromatic ring-hydroxylating Rieske oxygenases (Pfam PF00848). The network was generated by the EFI-EST server with a Uniref90 filter, an E-value of 5, and a final alignment score of 85. Clusters containing at least one sequence of a functionally verified protein are numbered. The numbers are associated with the "Sequence Count Cluster Number" attribute bestowed by the EFI-EST server. Table S4 lists representative UniProt IDs from each cluster that are associated with proteins/genes with published activities. In general cluster 1 contains glycine betaine oxidative demethylase-like proteins; 2 contains naphthalene/biphenyl/benzene/polyaromatic dioxygenase-like proteins; 3 contains benzoate and orthohalobenzoate-like proteins; 4 contains gamma glutamylanillidine dioxygenase-like proteins; 6 contains TphA2 and other monoaryl carboxylate oxygenase-like proteins; 9 contains carnitine monooxygenase-like proteins; 12 contains choline monooxygenase-like proteins; 19 contains p-cumate dioxygenase-like proteins; and 45 contains 2-aminobenzesulfonate oxygenase-like proteins.

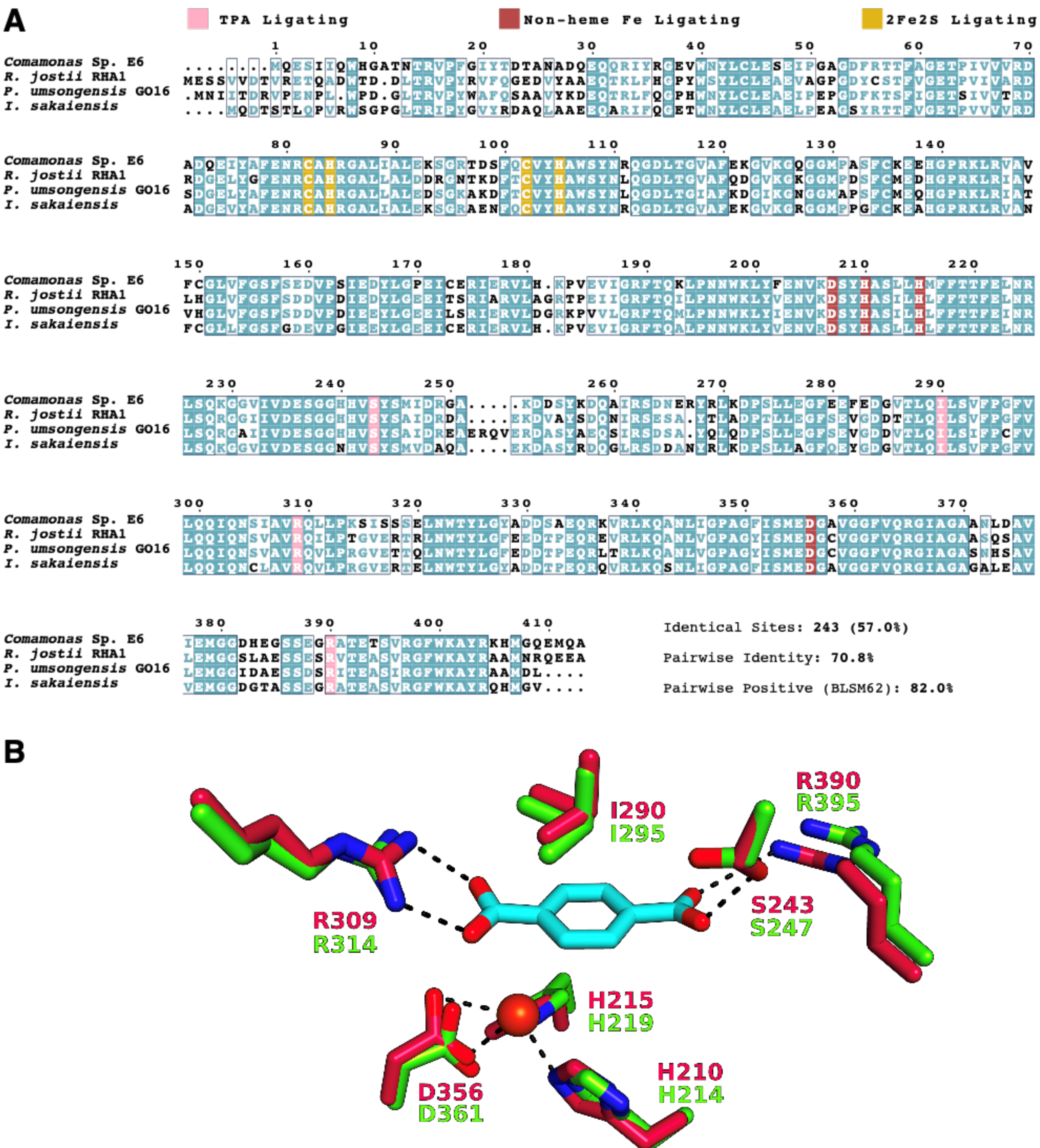

Figure S23. Multiple sequence alignment of TPADO α-subunits from known TPA metabolising bacteria and a structural overlay of the TPADO active sites from *Comamonas sp.* E6 and *Ideonella sakaiensis*.

A) Multiple sequence alignment of *TphA2* translated amino acid sequences from organisms known to utilize TPA as a sole carbon source (*Rhodococcus jostii* RHA1 (13), *Pseudomonas umsongensis* GO16 (14), *Ideonella sakaiensis* (15), and *Comamonas* sp. E6 (16)). Residues participating in TPA ligation (pink), non-heme Fe ligation(red), and 2Fe2S cluster ligation (gold) are highlighted. These residues are conserved across all four sequences, suggesting a structural conservation as well. B) Superposition of the active site residues coordinating TPA and non-heme Fe from *Comamonas* sp. E6 (red) with TPADO of the PET degrading bacterium *Ideonella sakaiensis* (green). The structure for *Is*TPADO was predicted using AlphaFold (17).

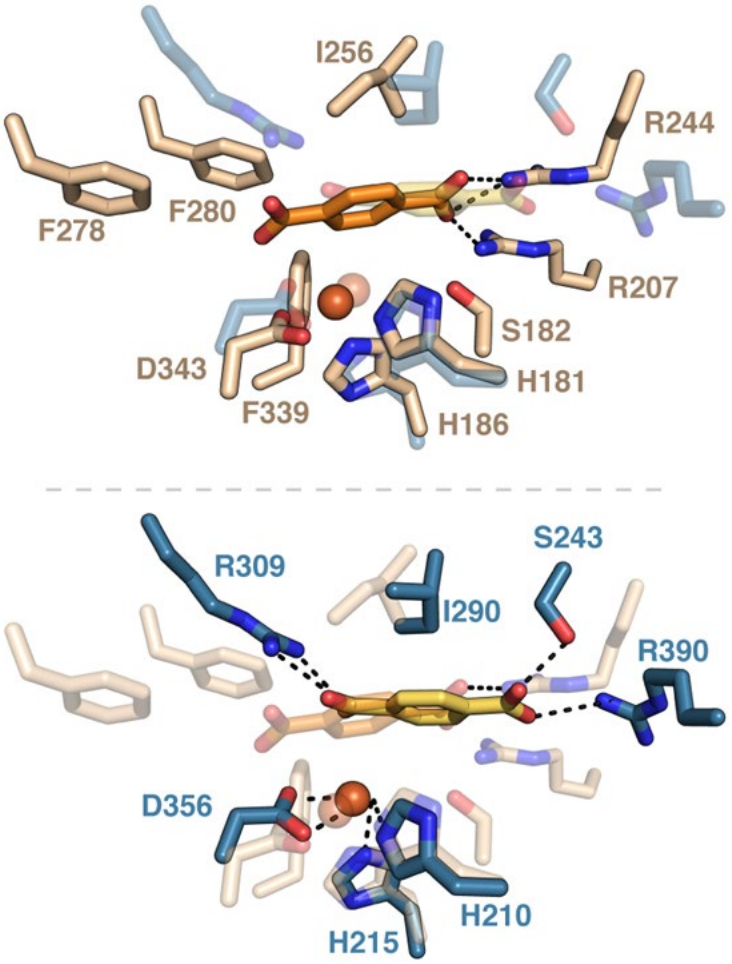

Figure S24. Superposition of the active site structures for the *ortho*-phthalate dioxygenase (PDO) from *Comamonas testosteroni* KF1 (PDB ID 7V28, carbon in steel blue, SI ref 18) and the TPADO from *Comamonas* strain E1 (PDB ID 7Q05, carbon in tan). Bound TPA is shown in the center of both structures. The top image has the PDO structure rendered at full opacity, with TPA in orange, while the bottom image has the TPAD at full opacity, with TPA in yellow. The α-subunits, on the whole, overlay poorly (RMSD = 3.21Å), and their primary sequences share 19% similarity. Dashed lines indicate electrostatic and/or hydrogen bonding interactions that anchor the TPA into the respective active sites. In PDO, one of the TPA carboxylates is located in a hydrophobic pocket, while the other carboxylate forms 3 interactions with R244 and R207. In TPADO, the TPA is multiply anchored via the carboxylates at both ends of the substrate. TPA dioxygenation by PDO occurred with 20% coupling to NADH consumption, with a 25-fold reduction in k_{cat}/K_M relative to the preferred *ortho*-phthalate substrate.

Table S1. HPLC analysis parameters obtained from standards†

Compound	retention time (min)	λ_{maxima}
DCD	11.7	270
NAD+	12.7	259
NADH	16.7	261, 320
PCA	19.7	259, 294, 331
TPA	23.3	240, 285
2OH-TPA	23.8	247, 325
$2NH_2$-TPA	22.5	228, 362
4NBA	25.5	261
4FBA	24.4	253, 294
4CBA	19.9	239, 284

†Retention time and spectral maximum are reported for DCD generated as the product of the TPADO catalyzed reaction, as no standards for this compound are available. It is assigned on the basis of its exact mass obtained by high resolution ESI-MS. (Stereochemistry not determined.)

Table S2: Crystallographic data and refinement statistics.

	Substrate-free state	TPA bound state	2-OH-TPA bound state
**Data collection**			
**Beamline**	DLS I03	DLS I03	DLS I03
**Space group**	$P6_1$	$P6_1$	$P6_1$
**Cell dimensions**			
**a, b, c (Å)**	220.8 / 220.8 / 84.1	219.7 / 219.7 / 83.0	220.3 / 220.3 / 83.2
**α, β, γ (°)**	90.0 / 90.0 / 120.0	90.0 / 90.0 / 120.0	90.0 / 90.0 / 120.0
**Resolution (Å)**	110.40 – 2.28 (2.44 – 2.28) [a]	190.28 – 2.08 (2.25 – 2.08)	190.80 – 1.95 (2.06 – 1.95)
**R_{merge} [%]**	23.9 (276.7)	16.7 (251.4)	10.9 (237.2)
**R_{pim} [%]**	5.3 (60.5)	3.7 (55.7)	2.7 (56.0)
**<I/σI>**	11.0 (1.4)	14.0 (1.6)	17.9 (1.4)
**Completeness (%)[b]**	94.3 (63.3)	96.0 (65.5)	96.4 (57.4)
**Redundancy**	21.1 (21.8)	21.3 (21.4)	17.3 (18.5)
**CC(1/2)**	0.998 (0.648)	0.999 (0.667)	0.999 (0.590)
**Refinement**			
**R_{work} / R_{free}**	18.0 / 22.5	16.8 / 21.4	16.5 / 20.2
**Ramachandran plot**			
**most favored [%]**	95.2	95.8	96.5
**allowed [%]**	4.4	4.0	3.4
**disallowed [%]**	0.3	0.2	0.1
**No. atoms**			
**protein**	13645	13914	13891
**water**	408	953	921
**ligands**	13	105	83
***B*-factors**			
**protein**	51.5	49.5	46.1
**water**	39.9	47.8	46.2
**ligands**	52.4	79.9	66.2
**R.m.s. deviations**			
**Bond lengths (Å)**	0.0112	0.0140	0.0136
**Bond angles (°)**	1.70	1.82	1.81
**pdb-code**	7Q04	7Q05	7Q06

[a]values in parentheses are for the highest-resolution shell
[b]ellipsoidal completeness

Table S3. Accurate mass data. Theoretical and observed masses for parent and fragment ions of NAD, NADH, TPA, and DCD for both MS and MSMS acquisition.

Compound	Ion	Fragment Loss	Acquisition	Expected mass	Observed Mass	Mass error (ppm)
**NAD**	$[M-H]^-$		MS	662.1018	662.1045	4.1
**NADH**	$[M-H]^-$		MS	664.1175	664.1163	1.8
**TPA**	$[M-H]^-$		MS	165.0193	165.02	4.2
**TPA**	$[M-H]^-$	CO_2	MS	121.0295	121.0301	5.0
**TPA**	$[M-H]^-$	CO_2	MSMS	121.0295	121.0301	5.0
**TPA**	$[M-H]^-$	$CO_2 + CO_2$	MSMS	77.0397	77.0403	7.8
**DCD**	$[M-H]^-$		MS	199.0248	199.0253	2.5
**DCD**	$[M-H]^-$	H_2O	MS	181.0142	181.015	4.4
**DCD**	$[M-H]^-$	H_2O	MSMS	181.0142	181.0115	14.9
**DCD**	$[M-H]^-$	$H_2O + CO_2$	MS	137.0244	137.0248	2.9
**DCD**	$[M-H]^-$	$H_2O + CO_2$	MSMS	137.0244	137.0248	2.9

Table S4. Uniprot IDs and inferred substrate type for sub clusters from PF00848 SSN

Sequence Count Cluster Number	Selected Representative Uniprot IDs	Verified substrate type(s)
**01**	Q1QYU7	glycine betaine
**02**	Q9X4W9, Q93UV3, Q8RTL4, Q8GJF2, Q7DJF2, Q75WN5, Q53122, Q51743, Q45695, P0ABR5, P0A110, O86899, M9PW10, D7RED3, A5W4F2	biphenyl, benzene, toluene, cumene, nitroarene, cinnamate, napthalene, polyaromatics
**03**	Q51601, P07769, O85673	benzoate, orthohalobenzoate, anthranilate
**04**	Q76KR6	γ-glutamaylanilide
**06**	Q84BZ3, Q3C1E3, O87618, O52379, A0A1X9WE59, Q83VL2, Q65AS6	terephthalate, salicylate, hydroxy-picolinate, anthranilate
**09**	D0C9N6	carnitine
**12**	O04121	choline
**19**	Q51974	p-cumate
**45**	Q9RBG5	2-aminobenzene sulfonate

SI References

1. Terrific Broth. *Cold Spring Harbor Protocols* 2006(1):pdb.rec8620.
2. Luo ML*, et al.* (2016) The CRISPR RNA-guided surveillance complex in Escherichia coli accommodates extended RNA spacers. *Nucleic Acids Research* 44(15):7385-7394.
3. Vonrhein C*, et al.* (2018) Advances in automated data analysis and processing within autoPROC, combined with improved characterisation, mitigation and visualisation of the anisotropy of diffraction limits using STARANISO. *Acta Crystallogr. Sect. A Found. Adv* 74:a360-a360.
4. Vagin A & Teplyakov A (1997) MOLREP: an automated program for molecular replacement. *Journal of applied crystallography* 30(6):1022-1025.
5. Waterhouse A*, et al.* (2018) SWISS-MODEL: homology modelling of protein structures and complexes. *Nucleic acids research* 46(W1):W296-W303.
6. Emsley P, Lohkamp B, Scott WG, & Cowtan K (2010) Features and development of Coot. *Acta Crystallographica Section D: Biological Crystallography* 66(4):486-501.
7. Murshudov, G. N., Skubak, P., Lebedev, A. A., Pannu, N. S., Steiner, R. A., Nicholls, R. A., Winn, M. D., Long, F., and Vagin, A. A. (2011) REFMAC5 for the refinement of macromolecular crystal structures. *Acta Crystallographica Section D* **67**, 355-367.
8. Williams CJ*, et al.* (2018) MolProbity: More and better reference data for improved all-atom structure validation. *Protein Science* 27(1):293-315.
9. Holm L (2020) DALI and the persistence of protein shape. *Protein Science* 29(1):128-140.
10. Gasteiger E*, et al.* (2005) Protein identification and analysis tools on the ExPASy server. *The Proteomics Protocols Handbook*:571-607.
11. Munck E, *et al.* (1984) Purification and characterization of the Rieske iron sulfur protein from *Thermus thermophilus*. *Journal of Biological Chemistry* 259(1):124-133.
12. Hantke GT, *et al.* (2011) FdC1, a novel ferredoxin capable of alternative electron partitioning, increases in conditions of acceptor limitation at photosystem. *Journal of Biological Chemistry* 286(1):50-59.
13. H. Hara, *et. al.*, Transcriptomic analysis reveals a bifurcated terephthalate degradation pathway in *Rhodococcus* sp. strain RHA1. *Journal of Bacteriology* 189, 1641-1647 (2007).
14. T. Narancic, *et al.*, Genome analysis of the metabolically versatile *Pseudomonas umsongensis* GO16: The genetic basis for pet monomer upcycling into polyhydroxyalkanoates. *Microb. Biotech*. 14, 2463-2480 (2021).
15. S. Yoshida, *et al.*, A bacterium that degrades and assimilates poly(ethylene terephthalate). *Science* 351, 1196-1199 (2016).
16. M. Sasoh, *et al.*, Characterization of the terephthalate degradation genes of *Comamonas* sp, strain E6. Appl. *Environ. Microbiol*. 72, 1825-1832 (2006)
17. A.W. Senior *et. al.,* Improved protein structure prediction using potentials from deep learning. *Nature*, 577(7792), 706–710 (2020)
18. J. K. Mahto, *et al*., Molecular insights into substrate recognitionand catalysis by phthalate dioxygenase from *Comamonas testosteroni*. *J. Biol. Chem*.(2021) ASAP.
